# The effect of transcranial direct current stimulation on static and dynamic posture control in the elderly: a systematic review and meta-analysis

**DOI:** 10.3389/fnagi.2025.1645962

**Published:** 2025-08-13

**Authors:** Yaru Wei, Peng Chen, Jianglong Zhan, Lulu Yin, Zhongqi Yu, Lin Wang

**Affiliations:** Key Laboratory of Exercise and Health Sciences of Ministry of Education, School of Exercise and Health, Shanghai University of Sport, Shanghai, China

**Keywords:** transcranial direct current stimulation, older adults, postural control, systematic review, meta-analysis

## Abstract

**Purpose:**

This systematic review and meta-analysis aimed to investigate the effects of transcranial direct current stimulation (tDCS) on static and dynamic postural control in older adults, with the goal of providing evidence-based support for tDCS interventions in fall prevention among the elderly.

**Methods:**

PubMed, Web of Science, Embase, Cochrane Library, Scopus and CNKI were searched from their inception to March 11, 2025, covering literature published in all languages. Eligible studies included randomized controlled trials or randomized crossover trials assessing the effects of tDCS on static or dynamic postural control in older adults. The methodological quality and risk of bias of included studies were assessed using the PEDro scale and the Cochrane Risk of Bias Tool, respectively. Meta-analysis was performed using Stata 14.0 with a random-effects model. Subgroup analyses and meta-regression were performed to explore potential moderators.

**Results:**

A total of 19 studies were included in the systematic review, of which 14 were subjected to meta-analysis. Compared to control conditions, tDCS significantly improved following outcomes in older adults, static postural stability index (APSI_static_: *p* < 0.001; MLSI_static_: *p* < 0.001; OSI_static_: *p* < 0.001), single-leg stance time (*p* = 0.004), center of pressure (COP) sway area during quiet standing (*p* = 0.044), COP path length (*p* = 0.03), dynamic postural stability index (APSI_dynamic_: *p* < 0.001; MLSI_dynamic_: *p* < 0.001; OSI_dynamic_: *p* < 0.001), Timed Up and Go test (TUGT; *p* = 0.003), and stride time variability during walking (*p* < 0.001). Subgroup analyses indicated that tDCS efficacy varied according to stimulation site and intervention duration. Meta-regression further revealed that the effect of tDCS on single-leg stance time was influenced by mean age.

**Conclusion:**

These findings suggested that tDCS can significantly improve static and dynamic postural control in older adults. However, due to the limited number of included studies and substantial heterogeneity observed in some analyses, the current conclusions require further validation through high-quality research. Based on the available evidence, it is recommended that future studies focus on the application of tDCS in fall-prevention interventions among older adults, in order to provide stronger evidence for its implementation in clinical practice.

**Systematic review registration:**

This systematic review was registered in PROSPERO (International Prospective Register of Systematic Reviews) (Unique Identifier: [registration number: CRD420251031377]). The protocol is publicly available at: [https://www.crd.york.ac.uk/PROSPERO/].

## 1 Introduction

Falls are among the leading causes of injury and even mortality in older adults, which is closely related to the age-related decline in postural control (Khow and Visvanathan, 2017; Yin et al., 2025a). Postural control relies on the integration of somatosensory, vestibular, and visual information, a process primarily governed by the central nervous system to coordinate appropriate motor responses (Riemann and Lephart, 2002; Ivanenko and Gurfinkel, 2018). With advancing age, the ability to integrate sensory inputs diminishes, accompanied by a decline in the efficiency of both sensory and motor systems. Moreover, the deterioration of brain function is considered a primary contributor to these changes (Goble et al., 2012; Lee and Kim, 2022; Parthasharathy et al., 2022). Multiple studies have further shown that, compared to younger individuals, older adults exhibit reduced neural network connectivity, cortical excitability, and brain activity during postural control tasks (Fjell et al., 2017; Liu et al., 2018). These age-related changes in brain function result in impairments in neural resource allocation and motor control. As a result, older adults often demonstrate slower motor response times (Frolov et al., 2020), delayed postural adjustments (Lin et al., 2004), and diminished multitasking capabilities (Srygley et al., 2009; Li et al., 2018; Campos et al., 2022), significantly increasing the risk of falls. Therefore, identifying effective interventions to enhance brain function in older adults is crucial for improving their quality of life and reducing the socioeconomic burden associated with falls.

Transcranial direct current stimulation (tDCS), a safe and easy-to-administer noninvasive brain stimulation technique, has been widely used to modulate brain function and cortical excitability (Zhan et al., 2023). In recent years, it has also been applied to improve postural control not only in younger adults or clinical populations (e.g., individuals with stroke or Parkinson’s disease), but also in older adults, who often experience age-related declines in balance and motor function. Several studies have reported that, compared to sham stimulation, active transcranial direct current stimulation (tDCS) can partially enhance static and dynamic postural control in older adults. For instance, Moon et al. (2015) demonstrated that administering 0.5 mA tDCS over the primary motor cortex (M1) across 15 sessions resulted in improved postural stability, as indicated by prolonged single-leg stance time and reduced postural sway. Similarly, Manor et al. (2016) found that a single session of 2 mA tDCS targeting the dorsolateral prefrontal cortex (DLPFC) led to increased gait speed, suggesting a potential enhancement in dynamic balance (Moon et al. 2015; Manor et al., 2016). However, conflicting results have also been reported. Craig and Doumas (2017) found that single-session tDCS targeting the motor cortex or cerebellum failed to improve COP path length during bipedal stance tasks in older adults. Some studies have even suggested that single-session tDCS targeting the dorsolateral prefrontal cortex increased stride length variability during walking in older adults (Orcioli-Silva et al., 2021). These inconsistent findings may be attributed to variations in stimulation parameters (e.g., site, intensity, and duration) and sample characteristics (e.g., average age). Due to the lack of independent analysis of these confounding factors, the effectiveness of tDCS in improving static and dynamic postural control in older adults remains inconclusive. Systematically summarizing and analyzing these heterogeneous findings is essential to fully understand the impact of tDCS on balance function in older adults.

Therefore, the aim of this study was to conduct a systematic review and subsequent meta-analysis to comprehensively evaluate the effects of tDCS on static and dynamic postural control in older adults and to explore potential moderating factors and optimal stimulation parameters, thereby providing evidence-based guidance for future research and clinical practice.

## 2 Materials and methods

The protocol was registered in the International Prospective Register of Systematic Reviews (PROSPERO) (registration number: CRD420251031377) and followed the preferred reporting items for systematic reviews and meta-analysis (PRISMA) guidelines (Page et al., 2021).

### 2.1 Search strategy

PubMed, Web of Science, Embase, The Cochrane Library, Scopus and China National Knowledge Infrastructure (CNKI) electronic databases were searched from inception until March 11, 2025. Additional searches of the reference lists of the included studies were also conducted to ensure the comprehensiveness of the results. Two researchers (YW and PC) decided on the search strategy through discussion and searched through a combination of subject terms and keywords, a third researcher (JZ) was consulted in case of disagreement. The search terms and strategies included: (Transcranial Direct Current Stimulation (Mesh) OR tDCS OR anodal transcranial direct current stimulation OR anodal tDCS* OR cathodal stimulation transcranial direct current stimulation OR cathodal tDCS* OR Transcranial Electrical Stimulation* OR non-invasive brain stimulation) AND (aged (Mesh) OR aging OR elderly OR older adult* OR old OR older) AND (Postural Balance (Mesh) OR postural control* OR balance OR static balance OR dynamic balance OR postural stability OR postural instability OR posture equilibrium* OR postural sway OR stand* OR stance OR mobility OR walk* OR gait OR sit-to-stand OR STS OR timed up and go OR TUG OR 6 min walk* OR 6 MW OR 6MWT) Minor adjustments were made to different databases. The full search strategy for each database is described in Supplementary material S1, item 2.1, Tables 1–6.

### 2.2 Inclusion criteria

The inclusion criteria were as follows: (1) Population: healthy older adults (aged ≥60 years) who were able to walk and stand independently, and had no history of neurological disorders or lower limb musculoskeletal injuries affecting gait or postural control; (2) Intervention: the experimental group received tDCS alone or in combination with functional training, while the control group received sham stimulation or functional training; (3) Outcomes: (i) static balance; (ii) dynamic balance, including gait function. Static balance was defined as the measurement of postural stability while the body remains stationary, whereas dynamic balance was defined as the measurement of stability during body or support surface movement (Gonçalves et al., 2022; Koshino and Kobayashi, 2023); (4) Study design: randomized controlled trials or randomized crossover trials; (5) Publication criteria: human studies published in any language were included.

### 2.3 Exclusion criteria

The following types of studies were excluded: (1) studies with low methodological quality, defined as a PEDro score below 4 (Cashin and McAuley, 2020); (2) studies lacking outcome data; (3) studies with questionable design or results; (4) conference abstracts, study protocols, systematic reviews, and case reports; (5) duplicate publications; (6) non-peer-reviewed studies; and (7) studies with unavailable data.

### 2.4 Study selection and data extraction

All randomized controlled trials or randomized crossover studies investigating the effects of tDCS on postural control in older adults were included. Studies published in languages other than Chinese or English were translated using DeepL Translator (Hu et al., 2023). The assessment protocol was required to include at least one of the following components: static balance ability, dynamic balance ability, or gait function. Static balance was assessed through single- or double-leg stance tests, with outcomes measured using parameters such as center of pressure (COP), balance duration, or stability index. Dynamic balance was evaluated using tasks such as double-leg stance, gait assessments (figure of 8 walk test, TUG, obstacle walk test, six-minute walk test), sit-to-stand tests, or other relevant instrument-based measures (e.g., biodex system). Outcome measures included variables such as stability index, balance duration, and gait-related parameters (e.g., walking speed, step cadence, stride length, stride time, and gait variability).

Data extraction was independently conducted by two researchers (YW and PC). In cases of disagreement, a third researcher (JZ) was consulted for resolution. The means and standard deviations of outcome variables were extracted at the follow-up time point closest to the end of the intervention. The following data were extracted from each included study: (1) general study information, including first author, publication year, study design, country, sample size (experimental/control group), and participant characteristics (age, sex); (2) intervention details, including stimulation intensity, duration, and frequency; (3) outcome measures: studies were required to include at least one outcome related to static balance, dynamic balance, or gait function. If target data were missing or unclear in the original publication, the corresponding author was contacted via email for clarification. Data from graphical representations were extracted using GetData Graph Digitizer. When results were reported as medians and interquartile ranges, means and standard deviations were estimated using the online tool^1^.

### 2.5 Assessment of quality and risk of bias

The methodological quality of the included studies was assessed using the PEDro scale, which consists of 11 dichotomous items (yes/no). Except for the first item, each “yes” response contributes one point, resulting in a total score out of 10. Scores of ≥6 were considered high quality, scores of 4–5 were considered moderate quality, and scores <4 were classified as low quality (Cashin and McAuley, 2020; Hu et al., 2023).

Additionally, the risk of bias in randomized controlled trials and randomized crossover trials was assessed using the Cochrane Risk of Bias tool (Sterne et al., 2011). This tool includes seven domains: (1) random sequence generation; (2) allocation concealment; (3) blinding of participants and personnel; (4) blinding of outcome assessment; (5) completeness of outcome data; (6) selective reporting; and (7) other sources of bias. The assessment was conducted independently by two reviewers. Each domain was rated as having a “low,” “high,” or “unclear” risk of bias, which was used to identify both individual and overall bias.

### 2.6 Level of evidence evaluation

The overall quality of evidence was summarized by two researchers (YW and PC) using the Summary of Findings table generated by the Grading of Recommendations Assessment, Development, and Evaluation (GRADE) approach (Shamseer et al., 2015). In cases of disagreement, a third researcher (JZ) was consulted to reach a consensus.

### 2.7 Statistical analysis

To evaluate the efficacy of transcranial direct current stimulation (tDCS) on postural control in older adults, a meta-analysis was conducted using a random-effects model in Stata 14.0. Standardized mean differences (SMDs) with corresponding 95% confidence intervals (CIs) were calculated. Subgroup analyses were performed based on task type, stimulation site, and intervention duration. Given that not all studies employed the same assessment methods, outcomes were grouped by measurement indicators. To ensure the reliability and validity of the findings, pooled meta-analysis was performed only when the number of extracted results for the same collection indicator was more than three (*n* ≥ 3). SMDs were used to estimate effect sizes (ES), with thresholds defined as follows: small effect (0.2–0.5), moderate effect (0.5–0.8), and large effect (>0.8).

Heterogeneity was assessed using the *I*^2^ statistic and the *Q* test. A *p*-value of < 0.05 was considered statistically significant. High heterogeneity was defined as *I*^2^ ≥ 75%, in which case the results should be interpreted with caution (Yin et al., 2025b). When substantial heterogeneity was present (*I*^2^ ≥ 75%), funnel plots were used to assess the risk of publication bias (Sterne et al., 2011). Sensitivity analysis was performed by sequentially excluding each included study to examine the robustness of the results (Hu et al., 2023).

In addition, meta-regression analyses were conducted using Stata 14.0 when at least three studies were available for a given outcome. These analyses were performed to explore potential moderators influencing the effects of tDCS. In the meta-regression, effect size was set as the dependent variable, and potential moderators—including publication year, geographical region, proportion of male participants, mean age, and stimulation intensity—were included as independent variables. Random-effects models were applied for all regression analyses.

## 3 Results

### 3.1 Search results

**Figure 1:**
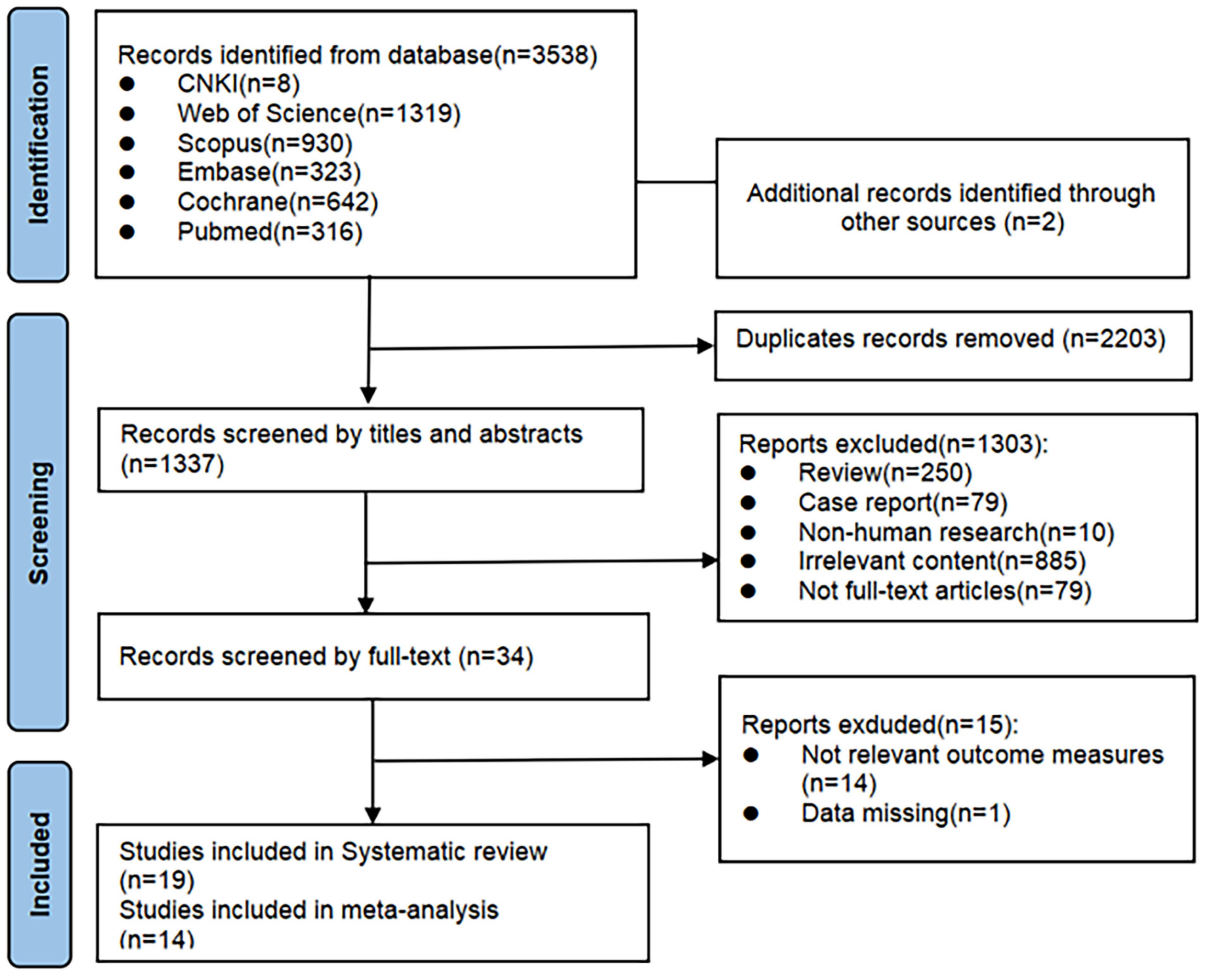
Flow chart of the screen outline of this systematic review and meta-analysis.

A total of 3,540 records were initially identified through database searching. After removing 2,203 duplicates using EndNote X9.1, 1,337 records remained. After screening titles and abstracts, 1,303 studies were excluded for not meeting the inclusion criteria. The full texts of the remaining 34 articles were reviewed, and 15 were excluded due to insufficient outcome data or unavailability of key results. Finally, 19 published randomized controlled trials were included in the systematic review. Of these, 14 studies were eligible for meta-analysis (see Figure 1).

### 3.2 Literature characteristics

Among the 19 included studies, 15 were randomized controlled trials (Moon et al., 2015; Ehsani et al., 2017; Kaminski et al., 2017; Yosephi et al., 2018; Mousavi Sadati and Rashidzadeh, 2019; Rostami et al., 2020; Shouhani et al., 2020; Clark et al., 2021; Orcioli-Silva et al., 2021; Yi et al., 2021; Correa et al., 2023; Lo et al., 2023; Parsaee et al., 2023; Rodrigues et al., 2023; Correa et al., 2024), and 4 were randomized crossover trials (Manor et al., 2016; Craig and Doumas, 2017; Zhou et al., 2018; Zhou et al., 2021). These studies were conducted across multiple countries in Asia, Europe, North America, and South America, with publication years ranging from 2015 to 2023. A total of 615 healthy older adults were included, with a mean age ranging from 61 to 90.75 years.

Of these, 10 studies (Son et al., 2015; Manor et al., 2016; Craig and Doumas, 2017; Ehsani et al., 2017; Yosephi et al., 2018; Mousavi Sadati and Rashidzadeh, 2019; Shouhani et al., 2020; Yi et al., 2021; Zhou et al., 2021; Parsaee et al., 2023) reported static balance outcomes; 18 studies (Son et al., 2015; Manor et al., 2016; Craig and Doumas, 2017; Ehsani et al., 2017; Kaminski et al., 2017; Yosephi et al., 2018; Zhou et al., 2018; Mousavi Sadati and Rashidzadeh, 2019; Rostami et al., 2020; Clark et al., 2021; Orcioli-Silva et al., 2021; Yi et al., 2021; Zhou et al., 2021; Correa et al., 2023; Lo et al., 2023; Parsaee et al., 2023; Rodrigues et al., 2023; Correa et al., 2024) reported dynamic balance outcomes; and 9 studies (Son et al., 2015; Manor et al., 2016; Zhou et al., 2018; Rostami et al., 2020; Clark et al., 2021; Orcioli-Silva et al., 2021; Yi et al., 2021; Lo et al., 2023; Correa et al., 2024) reported spatiotemporal gait parameters outcomes. Dynamic balance outcome measures included figure 8 walk time, Mini-balance evaluation systems test (Mini-BESTest) score, 6-min walk distance, anterior–posterior center of pressure (COP) path length, berg balance scale (BBS) score, anterior–posterior stability index (APSI), medial–lateral stability index (MLSI), overall stability index (OSI), mean balance time in the dynamic balance test (DBT), time spent during single−/dual-task timed up and go test (TUG), modified figure of 8 walk test time (MFEWT), 30-s chair stand test (30s-CST), 10-meter walk test (10MWT), 5-repetition sit-to-stand test (5STS), and number of steps in the MFEWT. Static balance outcomes included anterior–posterior COP path length, BBS score, APSI, MLSI, OSI, postural sway area and sway speed of COP during single−/dual-task standing, single-leg standing time, and functional reach test (FRT). Spatiotemporal gait parameters outcomes included typical, fastest, and obstacle walking speeds; single−/dual-task gait speed; stride time variability; stride length; step length; gait cadence; stance time; and gait speed under different task conditions.

All tDCS protocols used anodal stimulation, targeting the motor cortex, prefrontal cortex, or cerebellum. Stimulation intensities ranged from 0.5 to 2 mA (i.e., 0.5, 0.6, 1, 1.5, 1.98, and 2 mA), and intervention durations included immediate effects as well as 1, 2, 4, 5, 6, 8, and 12 weeks (see Supplementary material S1, item 2.2, Table 1 for details).

### 3.3 Quality and risk of bias evaluation results

The results of the methodological quality assessment are summarized in Table 1. The PEDro scores of the 19 included studies ranged from 4 to 10, with an average score of 7.63. One study (Lo et al., 2023) was rated as moderate quality, while the remaining studies (Son et al., 2015; Manor et al., 2016; Craig and Doumas, 2017; Ehsani et al., 2017; Kaminski et al., 2017; Yosephi et al., 2018; Zhou et al., 2018; Mousavi Sadati and Rashidzadeh, 2019; Rostami et al., 2020; Shouhani et al., 2020; Clark et al., 2021; Orcioli-Silva et al., 2021; Yi et al., 2021; Zhou et al., 2021; Correa et al., 2023; Parsaee et al., 2023; Rodrigues et al., 2023; Correa et al., 2024) were considered high quality.

**Table 1:** PEDro ratings for included studies.

Author, year	0	1	2	3	4	5	6	7	8	9	10	Total score
Clark et al. (2021)	Yes	Yes	Yes	Yes	Yes	Yes	Yes	Yes	Yes	Yes	Yes	10
Correa et al. (2023)	Yes	Yes	Yes	Yes	Yes	Yes	No	Yes	Yes	Yes	Yes	9
Correa et al., (2024)	Yes	Yes	Yes	Yes	Yes	Yes	Yes	Yes	Yes	Yes	Yes	10
Craig and Doumas (2017)	Yes	Yes	Yes	Yes	Yes	Yes	No	Yes	Yes	Yes	Yes	9
Ehsani et al. (2017)	Yes	Yes	No	Yes	Yes	Yes	No	Yes	Yes	Yes	Yes	7
Kaminski et al. (2017)	Yes	Yes	No	Yes	No	No	No	Yes	Yes	Yes	Yes	6
Lo et al. (2023)	Yes	Yes	Yes	No	Yes	Yes	No	No	No	No	No	4
Manor et al. (2016)	Yes	No	Yes	Yes	Yes	Yes	No	Yes	Yes	Yes	Yes	8
Shouhani et al. (2020)	Yes	Yes	No	Yes	Yes	No	No	Yes	Yes	Yes	Yes	7
Moon et al. (2015)	Yes	Yes	No	Yes	No	No	No	Yes	Yes	Yes	Yes	6
Parsaee et al. (2023)	Yes	Yes	No	Yes	Yes	No	No	Yes	Yes	Yes	Yes	7
Orcioli-Silva et al. (2021)	Yes	Yes	No	Yes	Yes	No	Yes	Yes	Yes	Yes	Yes	8
Rodrigues et al. (2023)	Yes	Yes	Yes	Yes	Yes	No	No	Yes	Yes	Yes	Yes	8
Rostami et al. (2020)	Yes	Yes	No	Yes	Yes	Yes	No	Yes	Yes	Yes	Yes	8
Mousavi Sadati and Rashidzadeh (2019)	Yes	Yes	No	Yes	Yes	No	No	Yes	Yes	Yes	Yes	7
Yi et al. (2021)	Yes	Yes	No	Yes	Yes	No	Yes	Yes	Yes	Yes	Yes	8
Yosephi et al. (2018)	Yes	Yes	No	Yes	Yes	No	Yes	Yes	Yes	Yes	Yes	7
Zhou et al. (2018)	Yes	Yes	No	Yes	Yes	Yes	No	Yes	Yes	Yes	Yes	8
Zhou et al. (2021)	Yes	Yes	No	Yes	Yes	Yes	No	Yes	Yes	Yes	Yes	8

0: eligibility criteria; 1: random allocation; 2: concealed allocation; 3: baseline comparability; 4: participants blinding; 5: therapists blinding; 6: assessors blinding; 7: follow-up > 85%; 8: intended treat; 9: between-group comparisons; 10: point measures and variability.

**Figure 2:**
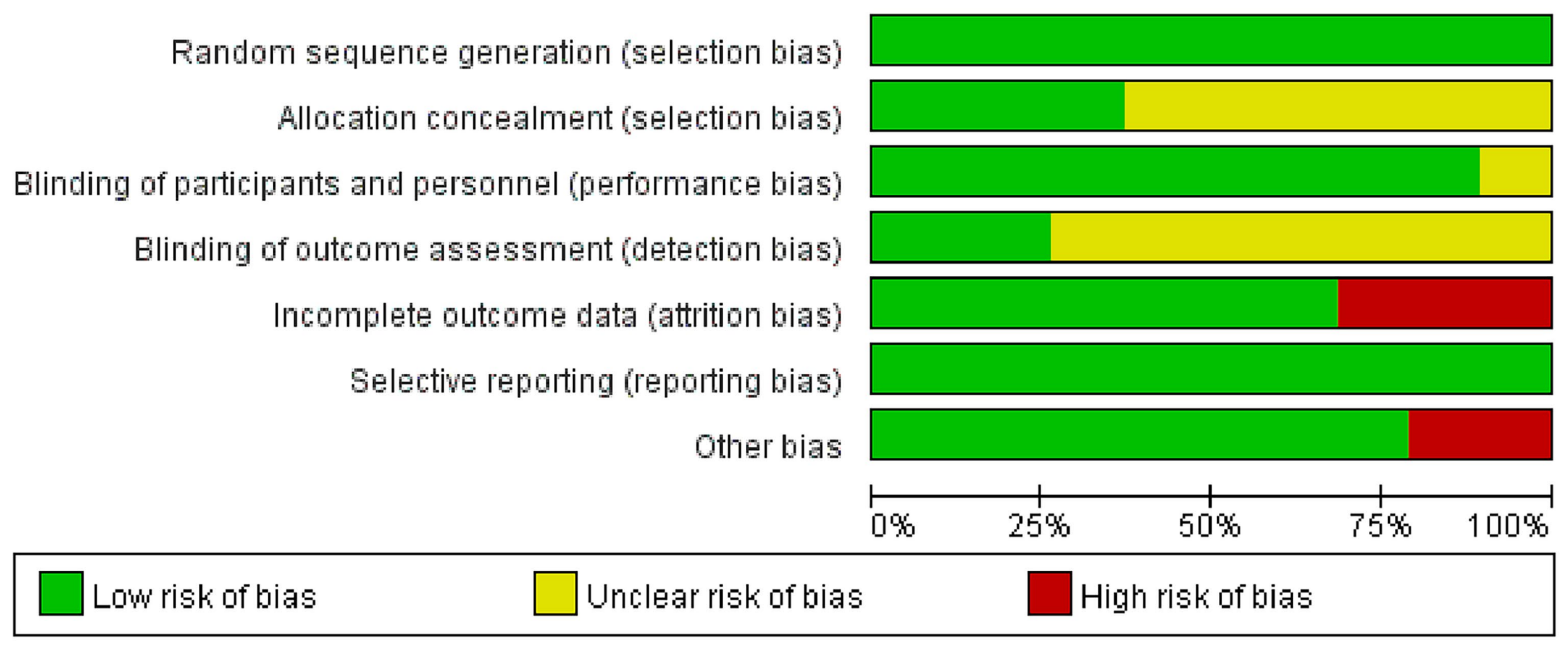
Risk of publication bias analysis of the included literature.

**Figure 3:**
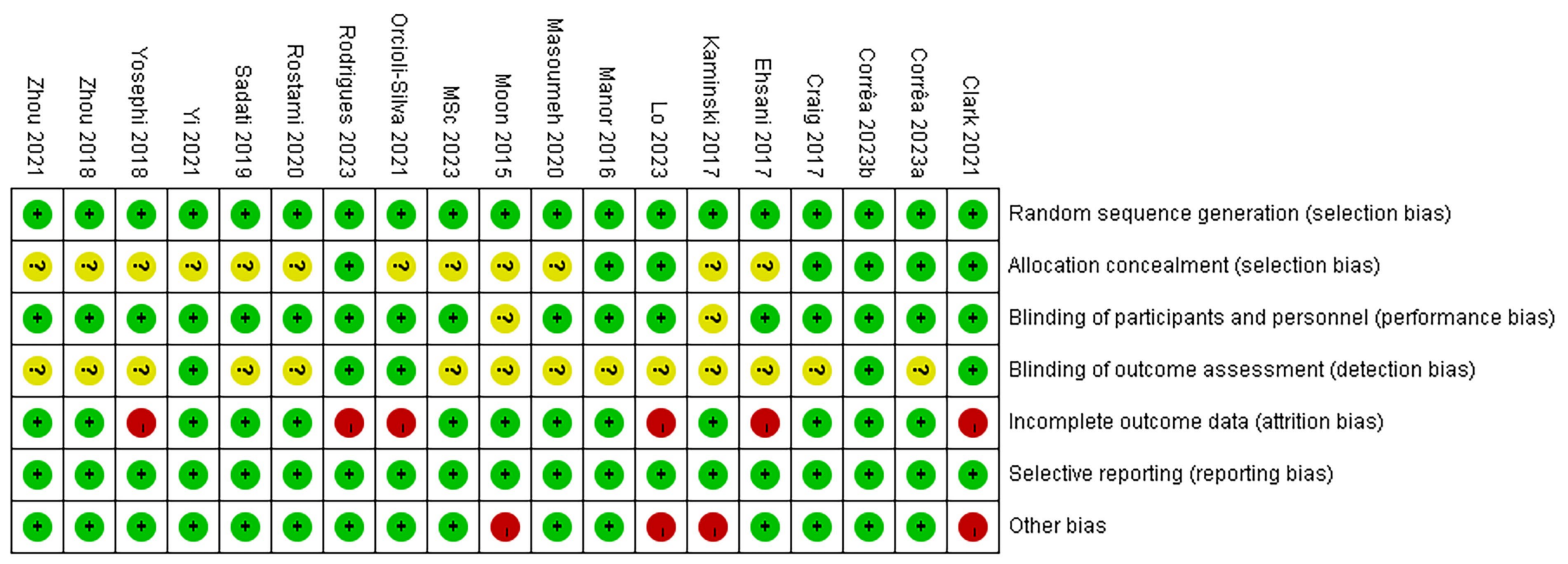
Risk of publication bias summary of the included literature.

The results of the risk of bias assessment are illustrated in Figure 2. Among the 19 studies, 7 (Ehsani et al., 2017; Clark et al., 2021; Yi et al., 2021; Correa et al., 2023; Lo et al., 2023; Rodrigues et al., 2023; Correa et al., 2024) reported specific methods for random sequence generation, whereas the others (Son et al., 2015; Manor et al., 2016; Craig and Doumas, 2017; Kaminski et al., 2017; Yosephi et al., 2018; Zhou et al., 2018; Mousavi Sadati and Rashidzadeh, 2019; Rostami et al., 2020; Shouhani et al., 2020; Orcioli-Silva et al., 2021; Zhou et al., 2021; Parsaee et al., 2023) only mentioned “randomly” without detailing the method. Allocation concealment was performed in 7 studies (Manor et al., 2016; Craig and Doumas, 2017; Clark et al., 2021; Correa et al., 2023; Lo et al., 2023; Rodrigues et al., 2023; Correa et al., 2024), while the remaining studies (Son et al., 2015; Ehsani et al., 2017; Kaminski et al., 2017; Yosephi et al., 2018; Zhou et al., 2018; Mousavi Sadati and Rashidzadeh, 2019; Rostami et al., 2020; Shouhani et al., 2020; Orcioli-Silva et al., 2021; Yi et al., 2021; Zhou et al., 2021; Parsaee et al., 2023) did not report this information and were therefore assessed as having unclear risk. Regarding blinding, 4 studies (Mousavi Sadati and Rashidzadeh, 2019; Shouhani et al., 2020; Parsaee et al., 2023; Rodrigues et al., 2023) used single-blinding, 11 studies (Manor et al., 2016; Craig and Doumas, 2017; Ehsani et al., 2017; Yosephi et al., 2018; Zhou et al., 2018; Rostami et al., 2020; Orcioli-Silva et al., 2021; Yi et al., 2021; Zhou et al., 2021; Lo et al., 2023; Correa et al., 2024) adopted double-blinding, and 2 studies (Clark et al., 2021; Correa et al., 2023) reported triple-blinding. Two studies (Son et al., 2015; Kaminski et al., 2017) did not mention blinding methods. Attrition was reported in 6 studies (Ehsani et al., 2017; Yosephi et al., 2018; Clark et al., 2021; Orcioli-Silva et al., 2021; Lo et al., 2023; Rodrigues et al., 2023), of which 5 (Ehsani et al., 2017; Yosephi et al., 2018; Clark et al., 2021; Lo et al., 2023; Rodrigues et al., 2023) provided specific reasons for participant withdrawal, while 1 study (Orcioli-Silva et al., 2021) did not. In addition, 4 studies (Son et al., 2015; Kaminski et al., 2017; Clark et al., 2021; Lo et al., 2023) were considered to have a high risk of other biases due to small sample sizes. Detailed information is presented in Figures 2, 3.

### 3.4 Meta-analysis

#### 3.4.1 Static balance

Static balance was quantified in two studies (Ehsani et al., 2017; Yosephi et al., 2018) using postural stability index obtained during double-leg standing. A meta-analysis was conducted based on directional postural stability index, as presented in Table 2. The pooled results demonstrated that, compared to the control group, tDCS exerted significant effects on APSI_static_ (SMD = −4.345, 95% CI: −4.846 to −3.844, *p* < 0.001), MLSI_static_ (SMD = −3.169, 95% CI: −3.584 to −2.753, *p* < 0.001), and OSI_static_ (SMD = −2.338, 95% CI: −2.736 to −1.940, p < 0.001), all indicating large effect sizes. Subgroup analyses revealed that both immediate and long-term (i.e., those lasting more than 2 weeks) tDCS, when applied to the cerebellum or M1 region, significantly enhanced directional postural stability index in older adults (Table 2). Forest and funnel plots related to the quantitative analysis are, respectively, provided in Supplementary material S1, item 1.1, Figures 1–3, item 1.2, Figures 1–3.

**Table 2:** Quantitative pooling results for static balance.

Outcomes	*n* of included results	Results			Subgroup analysis, *p*			Sensitivity analysis
		SMD (95% CI)	*p*	*I*^2^ (*p* for *I*^2^)	Stimulus location	Duration of intervention	Type of task	
APSI_static_	9	−4.532 (−5.262, −3.801)	**<0.001**	51.1% (0.038)	C: **<0.001** M1: **<0.001**	I: **<0.001** LT: **<0.001**	NA	Stable
MLSI_static_	9	−3.520 (−4.350, −2.690)	**<0.001**	73.7% (<0.001)	C: **<0.001** M1: **<0.001**	I: **<0.001** LT: **<0.001**	NA	Stable
OSI_static_	9	−3.327 (−4.819, −1.834)	**<0.001**	92.4% (<0.001)	C: **0.012** M1: **<0.001**	I: 0.058 LT: **<0.001**	NA	Stable
OLST	6	0.648 (0.203, 1.093)	**0.004**	53.2% (0.058)	C: **<0.001** M1: 0.242	I: 0.05 LT: **0.008**	NA	Stable
COP sway area	8	−0.663 (−1.308, −0.019)	**0.044**	94.9% (<0.001)	DLPFC: **0.047** M1: 0.172 DLPFC+M1: 0.639	NA	ST: 0.506 DT: 0.06	Unstable
COP path length	4	−0.924 (−1.757, −0.091)	**0.03**	79.6% (0.002)	C: **0.046** M1: 0.357	NA	NA	Unstable
COP sway velocity	6	−0.177 (−0.371 ~ 0.16)	0.073	39.6% (0.142)	DLPFC: 0.051 M1: 0.841 DLPFC+M1: 0.213	NA	ST: 0.347 DT: 0.217	Unstable

APSI, anterior–posterior stability index; MLSI, medial–lateral stability index; OSI, overall stability index; OLST, one-leg standing time; COP, center of pressure; C, cerebellum; M1, motor cortex; DLPFC, dorsolateral prefrontal cortex; I, immediate; LT, long term; ST, single task; DT, dual task; NA, not applicable. Bold values denote statistically significant differences (*p* < 0.05).

**Figure 4:**
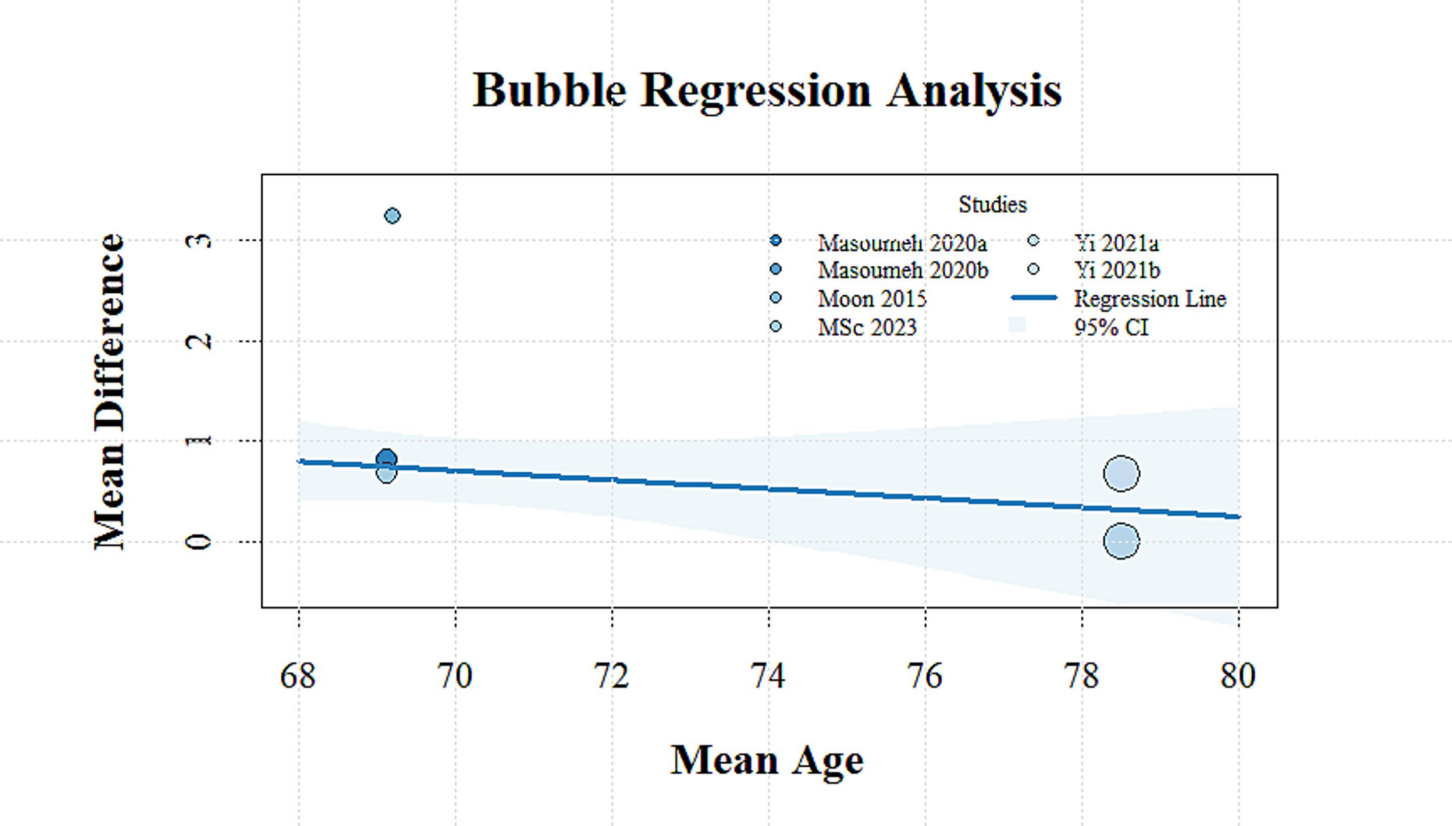
Bubble plot showing the relationship between mean age and mean difference in SLS time across studies.

Static balance was assessed in four studies (Son et al., 2015; Shouhani et al., 2020; Yi et al., 2021; Parsaee et al., 2023) using single-leg standing time. As presented in Table 2, the pooled results demonstrated that tDCS significantly increased single-leg standing time in older adults compared to the control group (SMD = 0.648, 95% CI: 0.203–1.093, *p* = 0.004), indicating a moderate effect size. Subgroup analyses revealed that tDCS targeting the cerebellum or administered as long-term stimulation significantly enhanced single-leg standing time, whereas no significant effects were observed with M1 stimulation or immediate stimulation (Table 2). Forest and funnel plots corresponding to the quantitative analysis are, respectively, provided in Supplementary material S1, item 1.1, Figure 4, item 1.2, Figure 4.

Static balance was evaluated in three studies (Manor et al., 2016; Craig and Doumas, 2017; Zhou et al., 2018) using COP parameters. As presented in Table 2, it was reported by Manor et al. (2016) and Zhou et al. (2021) that, compared to the control group, tDCS significantly reduced the COP sway area during eyes-open double-leg stance in older adults (SMD = −0.663, 95% CI: −1.308 to −0.019, *p* = 0.044), which represented a moderate effect size. Subgroup analyses indicated that tDCS applied to the prefrontal cortex significantly reduced COP sway area, while stimulation over the M1 region or combined M1 and prefrontal cortex showed no significant effects (Table 2). The effect of tDCS on anterior–posterior COP path length during quiet stance was examined by Craig and Doumas (2017), and as shown in Tables 2, a significant reduction was observed during eyes-open double-leg stance compared to the control group (SMD = −0.924, 95% CI: −1.757 to −0.091, *p* = 0.03), indicating a large effect size. Subgroup analyses revealed significant effects with cerebellar stimulation, while no significant effect was found for M1 stimulation. Additionally, Zhou et al. (2021) evaluated the effects of tDCS on COP sway velocity during eyes-open double-leg stance under both single- and dual-task conditions. However, no significant effect was observed (SMD = −0.177, 95% CI: −0.371 to 0.16, *p* = 0.073), and no significant subgroup differences were identified based on stimulation site or task type (Table 2). Forest and funnel plots for the quantitative analysis are, respectively, presented in Supplementary material S1, item 1.1, Figures 5–7, item 1.2, Figures 5–7.

#### 3.4.2 Dynamic balance

Dynamic balance was assessed in two studies (Ehsani et al., 2017; Yosephi et al., 2018) using postural stability index during double-leg stance. As shown in Table 3, meta-analysis based on direction-specific postural stability index demonstrated that, compared with the control group, tDCS exerted significant effects on APSI_dynamic_ (SMD = −3.418, 95% CI: −4.264 to −2.572, *p* < 0.001), MLSI_dynamic_ (SMD = −3.940, 95% CI: −4.642 to −3.238, *p* < 0.001), and OSI_dynamic_ (SMD = −3.923, 95% CI: −4.724 to −3.121, *p* < 0.001), all representing large effect sizes. Subgroup analyses revealed that both immediate and long-term tDCS applied to the cerebellum or M1 regions significantly improved dynamic postural stability index in all directions (Table 3). Forest and funnel plots for the quantitative analysis are, respectively, presented in Supplementary material S1, item 1.1, Figures 8–10, item 1.2, Figures 8–10.

**Table 3:** Quantitative pooling results for dynamic balance.

Outcomes	*n,* reference	Results			Subgroup analysis, *p*			Sensitivity analysis
		SMD (95% CI)	*p*	*I*^2^ (*p* for *I*^2^)	Stimulus location	Duration of intervention	Type of task	
APSI_dynamic_	17	−3.418 (−4.264, −2.572)	**<0.001**	87.8% (<0.001)	C: **<0.001** M1: **<0.001**	I: **<0.001** LT: **<0.001**	NA	Stable
MLSI_dynamic_	17	−3.940 (−4.642, −3.238)	**<0.001**	77.8% (<0.001)	C: **<0.001** M1: **<0.001**	I: **<0.001** LT: **<0.001**	NA	Stable
OSI_dynamic_	17	−3.923 (−4.724, −3.121)	**<0.001**	84.1% (<0.001)	C: **<0.001** M1: **<0.001**	I: **<0.001** LT: **<0.001**	NA	Stable
TUGT	7	−0.401 (−0.662, −0.140)	**0.003**	0% (0.43)	C: **0.027** DLPFC: 0.241 M1: **0.08**	I: 0.264 LT: **0.003**	ST: **0.009** DT: 0.335	Stable

APSI, anterior–posterior stability index; MLSI, medial–lateral stability index; OSI, overall stability index; TUGT, time spent during timed up and go; C, cerebellum; M1, motor cortex; DLPFC, dorsolateral prefrontal cortex; I, immediate; LT, long term; NA, not applicable. Bold values denote statistically significant differences (*p* < 0.05).

In five studies (Zhou et al., 2018; Rostami et al., 2020; Yi et al., 2021; Parsaee et al., 2023; Rodrigues et al., 2023), the TUG test was used to evaluate dynamic balance. As shown in Table 3, the pooled results indicated that, compared with the control group, tDCS significantly reduced TUG completion time in older adults (SMD = −0.401, 95% CI: −0.662 to −0.140, *p* = 0.003), indicating a small effect size. Subgroup analyses showed that tDCS applied to the cerebellum or M1, as well as long-term stimulation, significantly reduced TUG times. However, no significant effects were found with prefrontal cortex stimulation or immediate tDCS stimulation. In addition, a significant reduction in TUG time was observed under single-task conditions, whereas no significant effect was found under dual-task conditions (Table 3). Forest and funnel plots for the quantitative analysis are, respectively, shown in Supplementary material S1, item 1.1, Figure 11, item 1.2, Figure 11.

### 3.5 Spatiotemporal gait parameters

Spatiotemporal gait parameters were assessed in three studies (Manor et al., 2016; Clark et al., 2021; Zhou et al., 2021) using walking speed as an outcome measure. As shown in Table 4, the pooled analysis indicated that tDCS had no significant effect on walking speed in older adults compared with the control group (SMD = 0.332, 95% CI: −0.087 to 0.750, *p* = 0.12). Subgroup analyses revealed that immediate tDCS significantly improved walking speed, whereas no significant effects were observed with stimulation of the cerebellum, M1, or combined cerebellum-M1 regions, nor with long-term stimulation. Furthermore, no significant effects of tDCS were found on walking speed under either single-task or dual-task conditions (Table 4). Forest and funnel plots of the quantitative analyses are, respectively, shown in Supplementary material S1, item 1.1, Figure 12, item 1.2, Figure 12.

**Table 4:** Quantitative pooling results for spatiotemporal gait parameters.

Outcomes	*n,* reference	Results			Subgroup analysis, *p*			Sensitivity analysis
		SMD (95% CI)	*p*	*I*^2^ (*p* for *I*^2^)	Stimulus location	Duration of intervention	Type of task	
Walking speed	11	0.332 (−0.087, 0.750)	0.12	87.7% (<0.001)	DLPFC: 0.217 M1: 1 DLPFC+M1: 0.452	I: **0.042** LT: 0.273	ST: 0.442 DT: 0.159	Stable
Stride time variability	7	−0.271 (−0.419, −0.122)	**<0.001**	0% (0.608)	DLPFC: **0.021** M1: 0.188 DLPFC+M1: **0.022**	NA	ST: **<0.001** DT: 0.238	Stable

M1, motor cortex; DLPFC, dorsolateral prefrontal cortex; I, immediate; LT, long term; ST, single task; DT, dual task; NA, not applicable. Bold values denote statistically significant differences (*p* < 0.05).

Spatiotemporal gait parameters was further assessed in two studies (Orcioli-Silva et al., 2021; Zhou et al., 2021) using stride time variability. As presented in Table 4, the pooled results demonstrated that tDCS significantly reduced stride time variability during walking in older adults (SMD = −0.271, 95% CI: −0.419 to −0.122, *p* < 0.001). Subgroup analysis revealed that tDCS applied to the prefrontal cortex or the combined prefrontal-M1 regions significantly reduced stride time variability during walking in older adults, whereas stimulation of the M1 region alone did not yield significant effects. Additionally, a significant reduction in stride time variability was found under single-task conditions, while no significant effects were detected under dual-task walking conditions (Table 4). Forest and funnel plots are, respectively, provided in Supplementary material S1, item 1.1, Figure 13, item 1.2, Figure 13.

### 3.6 Meta-regression

Meta-regression analysis was conducted to examine potential confounding variables influencing the magnitude of outcome effects, including the stimulation site, publication year, geographic region, proportion of male participants, mean age, and stimulation intensity. None of the covariates demonstrated a statistically significant effect on TUGT performance (*p* > 0.05). A negative association was observed between improvements in single-leg standing time and mean age (*β* = −0.09, 95% CI: −0.18 to −0.01, *p* = 0.041) (Figure 4), whereas no significant relationships were identified with the remaining covariates (*p* > 0.05). Further details are provided in Supplementary material S1, item 2.3, Table 1.

### 3.7 Sensitivity analysis

To further examine the sources of heterogeneity, a sensitivity analysis was performed on various outcome measures by systematically removing each individual studies. The results are shown in Tables 2–4. Except for COP sway area, path length, and sway velocity, all other outcome measures remained consistent, indicating that the findings of the meta-analysis are relatively stable. Further details are provided in Supplementary material S1, item 1.3, Figures 1–13.

### 3.8 Level of evidence

According to the GRADE system, the overall certainty of evidence was rated as low across all outcomes. A detailed assessment of evidence quality is provided in Supplementary materials S1, item 2.4, Table 1. The primary reasons for downgrading the evidence were risk of bias, heterogeneity, and imprecision.

## 4 Discussion

This current study included 19 randomized clinical trials that systematically evaluated the effects of tDCS on postural control in healthy older adults and showed that tDCS significantly reduced static and dynamic stability index, COP swing area, COP path length, spent time in TUG, and stride time variability compared to the control group, implying that tDCS can significantly enhance static and dynamic postural control.

### 4.1 Main findings

Postural control relies on the integrated coordination of the sensory, central nervous, and motor systems (Alfieri et al., 2012; Ivanenko and Gurfinkel, 2018; Anagnostou and Hepple, 2020). Among these, the central nervous system (CNS) plays a pivotal role as the core processor of information integration and regulation. Consequently, an increasing number of studies have emphasized the importance of targeting adaptive changes in cortical motor and sensory regions for intervention. tDCS have shown promising therapeutic potential in this regard. The findings of the present study demonstrate that tDCS significantly improves both static and dynamic postural control in older adults, thereby reinforcing previous research conclusions. These findings suggest that tDCS is a promising strategy to enhance postural control, which may contribute to fall risk reduction in older adults (Gschwind et al., 2013; Cuevas-Trisan, 2019; Colón-Emeric et al., 2024).

In the included studies, various cortical targets were selected with the aim of improving postural control by targeting distinct but functionally relevant brain regions. These were grouped into three main areas for subgroup analysis: the motor cortex and cerebellum, which are primarily involved in motor control and sensorimotor integration (Andersen and Buneo, 2002; Morton and Bastian, 2004), and the dorsolateral prefrontal cortex (DLPFC), a key region implicated in cognitive and executive functions that contribute to balance regulation (Yogev-Seligmann et al., 2008). The motor cortex plays a central role in voluntary movement execution and muscle coordination essential for maintaining stability (Andersen and Buneo, 2002), while the cerebellum integrates sensory feedback and fine-tunes motor commands to support postural adjustments (Morton and Bastian, 2004). The DLPFC supports attentional control, executive function, and dual-task processing, which are particularly important for maintaining balance under cognitively demanding conditions (Yogev-Seligmann et al., 2008). Some studies have suggested that electrode placement may influence the efficacy of tDCS (Schneider et al., 2021). Accordingly, subgroup analyses were conducted based on stimulation targets. An interesting finding was that stimulation over the DLPFC significantly reduced both center of pressure (COP) sway area and stride time variability, whereas stimulation over the motor cortex did not. The DLPFC is well known as a key region involved in cognitive and executive functions. With advancing age, connectivity between DLPFC regions tends to decline (Tucker and Stern, 2011). Several studies have reported improvements in decision-making, memory, and movement accuracy during motor tasks following stimulation of this region (Hecht et al., 2010; Reis and Fritsch, 2011; Metuki et al., 2012). Given that cognitive function plays a critical role in postural control, these results appear to be plausible. For instance, Zhou et al. (2015) observed improved postural control during dual-task performance (i.e., walking while performing mental arithmetic) in older adults following tDCS targeting the prefrontal cortex. While our analysis suggests potential limitations of single-area M1 stimulation, dual-site stimulation (e.g., DLPFC + M1) failed to show significantly enhanced balance effects compared to M1-only protocols. This may reflect either insufficient network engagement or methodological factors such as stimulation parameters and outcome measure sensitivity. Therapeutic interventions aimed at enhancing postural control in the elderly should not focus solely on motor areas; instead, targeting cognitive function may also yield beneficial effects on postural control.

Furthermore, stimulation targeting the DLPFC and the cerebellum was found to significantly improve single-leg standing time, whereas stimulation over the motor cortex did not yield similar effects. The cerebellum plays a crucial role in motor control, including limb coordination, postural control and balance, gait, and other bodily movements (Morton and Bastian, 2004). Specifically, the cerebellar vermis is one of the key regions responsible for balance and posture regulation, while the cerebellar white matter tracts serve as major conduits connecting the cerebellum to other brain areas. Cerebellar tDCS may enhance the function of the vermis or white matter tracts by increasing the activity of Purkinje cells (Celnik, 2015). Indeed, tDCS has been shown to facilitate cerebellar connectivity with other brain regions and to augment cerebellar influence on the motor cortex, vestibular system, midbrain, and additional neural networks (Priori et al., 2014; Rahmati et al., 2014). Therefore, tDCS may contribute to improved motor and balance performance by compensating for age-related structural changes in cerebellar regions among older adults. Taken together, these findings suggest that enhancing balance function may require stimulation targeted at brain regions selected based on their functional relevance, rather than relying solely on traditional motor areas.

A subgroup analysis was also conducted based on the duration of the intervention. The results indicated that both single-session and long-term tDCS interventions exerted positive effects on static and dynamic postural stability index. A previous study demonstrated that even a single session of tDCS enhanced the overall stability index in patients with post-stroke hemiparesis (Sohn et al., 2013). However, it is noteworthy that although a single session of tDCS may improve postural stability in older adults under controlled laboratory conditions, such effects are typically short-lived, lasting only 30 to 60 min (Vargas et al., 2018), and are therefore not suitable for clinical application. Earlier evidence has shown that tDCS induces membrane depolarization, thereby facilitating the effects of subsequent therapeutic stimulation (Stagg and Nitsche, 2011). Sustained physical exercise has also been reported to significantly affect synaptic activity and neuroplasticity related to physical functioning in healthy adults (Knaepen et al., 2010). Combining tDCS with motor tasks may thus offer greater benefits for the long-term improvement of postural control in older adults. Moreover, some studies have highlighted that the neurophysiological effects of tDCS differ between older and younger populations, with age-related delays in neural plasticity observed in the elderly (Fujiyama et al., 2014). Consequently, future research should consider applying tDCS prior to the performance of motor tasks to optimize its therapeutic effects.

### 4.2 Comparison with other studies

A previous study (Guo et al., 2020) systematically reviewed the effects of tDCS on postural control in older adults, and the results indicated that tDCS significantly improved static postural control and reduced TUG completion time. Overall, these findings are consistent with those of our study. It is important to note that this study included only a limited number of publications and did not distinguish between healthy individuals and those with neurological disorders, which considerably reduces the reliability and clinical applicability of the findings. The present study focused exclusively on healthy older adults and conducted subgroup analyses based on stimulation site, intervention duration, and testing tasks to further investigate the influence of different stimulation parameters. The findings also revealed that stimulation of the dorsolateral prefrontal cortex (DLPFC) led to more comprehensive improvements in postural control and gait function measures. It is noteworthy that tDCS only improved postural control and gait function under single-task conditions, but not under dual-task conditions.

A previous systematic review (Behrangrad et al., 2021) indicated that when tDCS was applied to the motor cortex and supplementary motor area, it improved postural control in healthy participants; however, when applied to the DLPFC, it only enhanced postural control under dual-task conditions. This finding is not consistent with our results. It is believed that differences in age may largely account for the discrepancies in findings. That study included healthy individuals aged 18 years and older. It is well established that substantial differences in brain structure and function exist among younger and older adults (Stewart et al., 2014), which may influence task performance and response to tDCS protocols, thereby increasing heterogeneity in outcomes. The regression analysis conducted in this study further supports the hypothesis that age-related differences may contribute to variability in outcomes. In particular, improvements in single-leg stance time were negatively correlated with the average age of participants across the included studies. Another possible explanation is that the subgroup analyses based on task type did not account for differences in stimulation sites; stimulation targeting regions other than the primary task-related areas may have been insufficient to improve dual-task postural control, thereby affecting the overall effect size.

### 4.3 Clinical significance

With population aging, falls have emerged as a significant public health concern, adversely affecting the health and quality of life of older adults. Deficits in postural control represent one of the primary risk factors for falls. Therefore, identifying safe and effective interventions to enhance postural control is essential, as improved balance may contribute to reduced fall risk and associated burdens, although this link was not directly assessed in the included studies (Cuevas-Trisan, 2019; Colón-Emeric et al., 2024). As a noninvasive neuromodulation technique, tDCS is characterized by high safety and operational simplicity. It can directly modulate cortical excitability and promote neuroplasticity, thereby enabling targeted intervention of the central postural regulatory network. According to the present findings, tDCS application significantly improved both static and dynamic postural control, as well as enhanced walking function in healthy older adults. This finding is of clinical significance, suggesting that tDCS may serve as an adjunctive therapeutic modality alongside exercise to enhance treatment efficacy in older adults at low risk of falls. Given its non-invasive nature and potential to improve balance, tDCS may serve as a supplementary approach for older adults who have limited access to or tolerance for physical exercise; however, further research is needed to clarify its comparative effectiveness.

Despite the promising therapeutic potential of tDCS, several challenges may hinder its widespread adoption in clinical or community settings. First, variability in individual responses to tDCS remains a key concern, particularly in older adults with heterogeneous neural aging profiles. Personalized protocols based on cognitive or neurophysiological markers may be needed to optimize outcomes. Second, the safe and effective administration of tDCS requires trained personnel and adherence to established safety guidelines, which may limit scalability outside specialized centers. Third, logistical issues such as cost, access to equipment, and the need for repeated sessions over time may reduce feasibility, especially in resource-limited settings. Moreover, long-term adherence among older adults may be affected by cognitive decline, comorbidities, or physical limitations. Regulatory approval and clinical guidelines for the use of tDCS in older populations are also still evolving, further complicating implementation. Future studies should address these barriers by evaluating home-based tDCS protocols, caregiver involvement, and integration with existing rehabilitation services to support safe and accessible delivery of tDCS in real-world contexts.

### 4.4 Strengths and limitations

This study possesses several notable strengths. First, to further elucidate the moderating role of intervention parameters on the effects of tDCS, subgroup analyses were conducted to examine the impact of different stimulation sites (e.g., motor cortex, cerebellum, and DLPFC) and intervention durations (immediate vs. long-term). Although stimulation intensity was not included in the subgroup analyses due to data limitations, we acknowledge its importance and recommend future studies to examine its influence. These findings provide more targeted references for tailoring individualized tDCS-based neurorehabilitation strategies, particularly for older adults with balance impairments or at high risk of falls. Second, a systematic and comprehensive search strategy was employed across multiple databases, without restrictions on publication date or language. This approach enabled the inclusion of a substantial number of randomized clinical trials, thereby ensuring adequate statistical power.

To be honest, this study has several limitations. First, the included primary studies reported only limited long-term observational outcomes, thereby precluding statistical synthesis of these trials. The importance of long-term follow-up is emphasized, as it is a critical factor for clinicians in the decision-making process. Then, several outcome measures were supported by a limited number of studies, which may have reduced the reliability of the findings. Furthermore, although stimulation site and intervention duration were considered potential moderators, stimulation intensity may also influence intervention outcomes. However, due to the limited number of studies using consistent intensity levels, a formal subgroup analysis was not feasible. Future research is encouraged to examine the dose–response relationship of stimulation intensity more systematically. Additionally, several included studies applied tDCS in conjunction with other interventions, such as physical exercise or balance training. In most cases, the control and intervention groups received the same conventional treatment, with the only variable being the presence of active versus sham tDCS. Therefore, the effect estimates primarily reflect the additional effect of tDCS. Given the substantial variability in the types, timing, and dosage of co-interventions, a formal subgroup analysis was not possible. Future meta-analyses with more standardized designs may further explore the interaction between tDCS and combined rehabilitation strategies. Finally, most included studies implemented short intervention durations (<6 weeks). Therefore, it remains unclear whether extended intervention durations could have yielded greater improvements in postural control among healthy older adults. Nevertheless, a strength of this study lies in its systematic evaluation of tDCS effects on both static and dynamic postural control in older adults, providing valuable insights for clinical application. Future studies need to determine whether increased training frequency and/or prolonged intervention duration result in greater improvements in postural control among older adults, and to assess the sustainability of such effects.

## 5 Conclusion

This systematic review and meta-analysis demonstrated that tDCS significantly improved both static and dynamic postural control in healthy older adults. Subgroup analyses further revealed that the intervention duration and stimulation site may be key moderators of the efficacy of tDCS. As a safe and non-invasive neuromodulation technique, tDCS shows promising potential in improving postural control in older adults, making it a potentially valuable tool to enhance balance, which may help mitigate fall risk in the elderly (Colón-Emeric et al., 2024). However, due to the limited number of available studies and substantial heterogeneity, the interpretation of current findings should be approached with caution. Future studies are recommended to conduct large-scale, multicenter randomized controlled trials to further verify the effects of tDCS and to explore its underlying neural mechanisms, thereby providing more robust evidence for its application in neuromodulatory strategies aimed at improving postural control, which may contribute to fall prevention in older adults.

## Data Availability

The original contributions presented in the study are included in the article/Supplementary material, further inquiries can be directed to the corresponding author.

## Footnotes

^1^ https://www.math.hkbu.edu.hk/~tongt/papers/median2mean.html
