# Supplementary material for: The effect of transcranial direct current stimulation on static and dynamic posture control in the elderly: a systematic review and meta-analysis
Source: Front Aging Neurosci. 2025 Aug 13;17:1645962. doi: 10.3389/fnagi.2025.1645962 (PMC12380813; doi:10.3389/fnagi.2025.1645962)
Supplement: Supplementary file 1 [file Data_Sheet_1.PDF]

Supplementary Material

1 Supplementary Figures

1.1 Forest plots

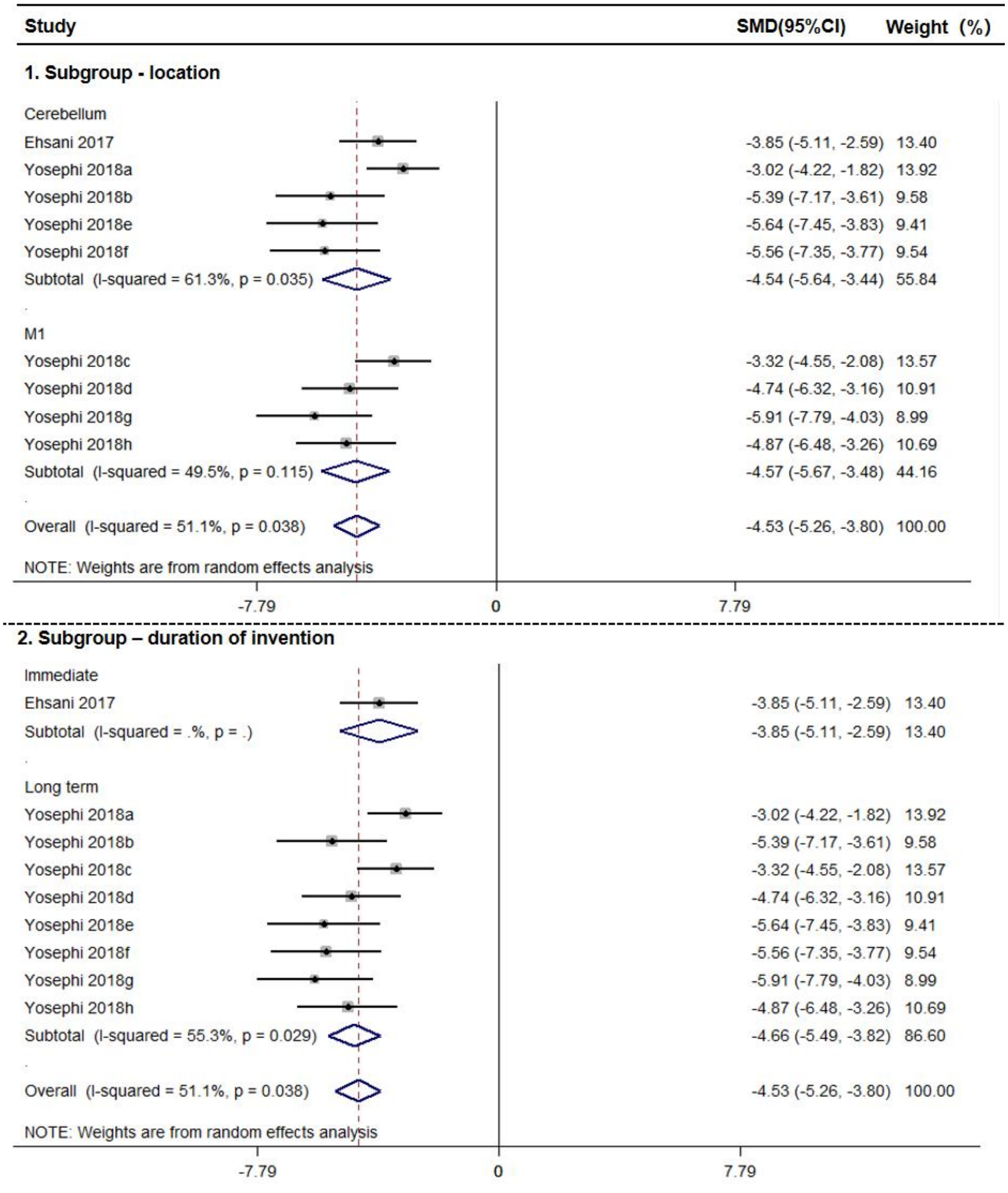

Figure 1. The forest plot results of Static balance-APSI comparing tDCS to Con.

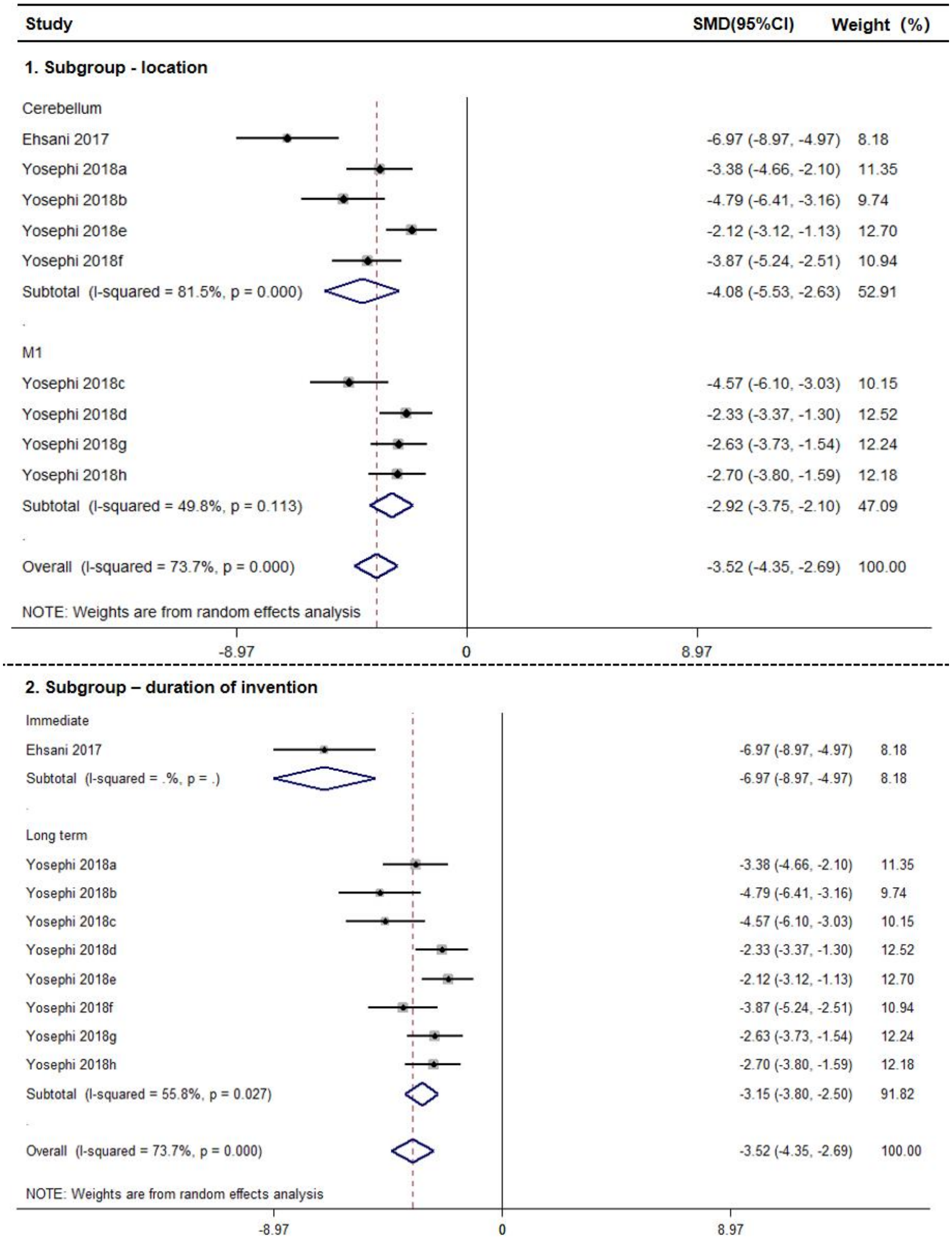

Figure 2. The forest plot results of Static balance-MLSI comparing tDCS to Con.

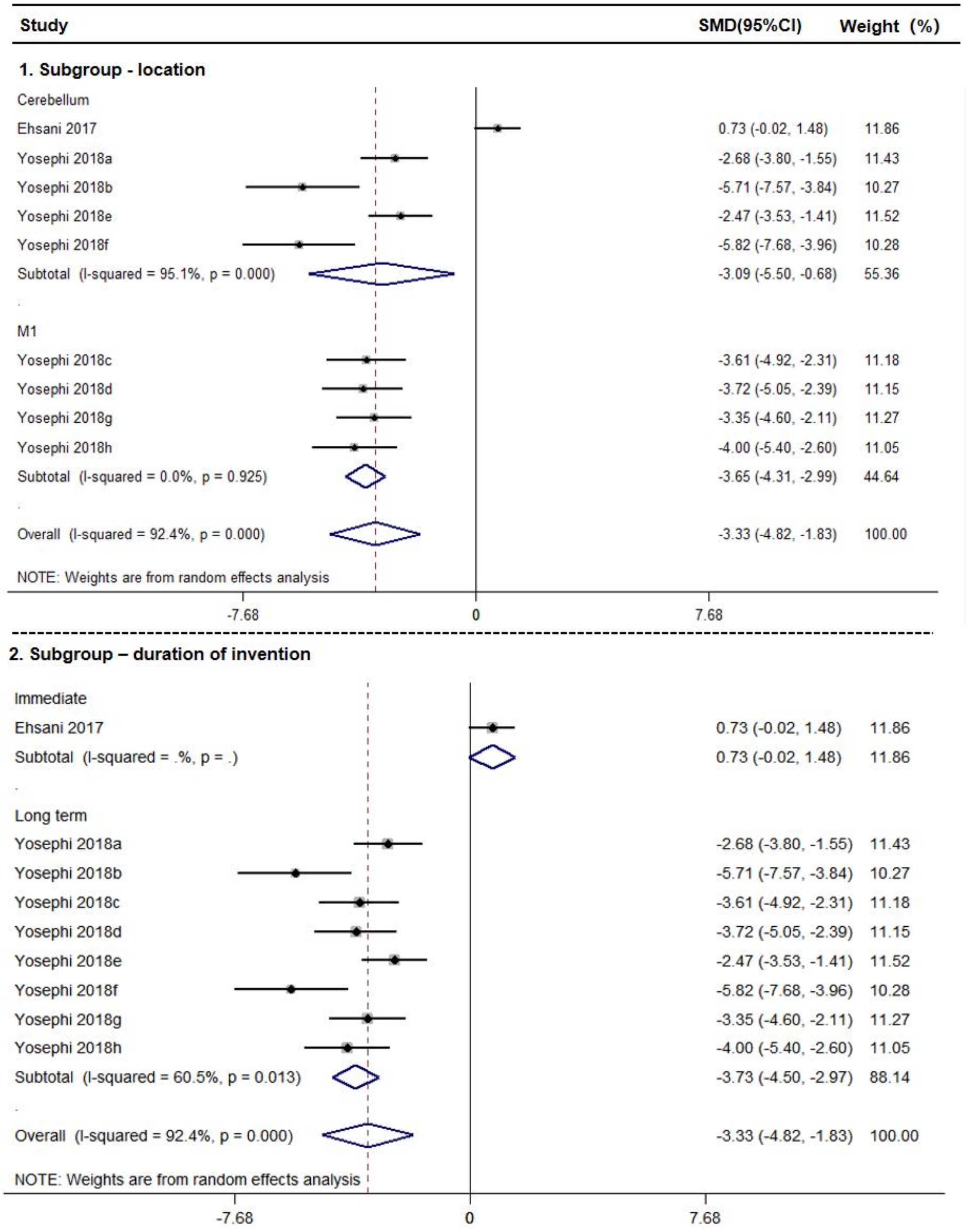

Figure 3. The forest plot results of Static balance-OSI comparing tDCS to Con.

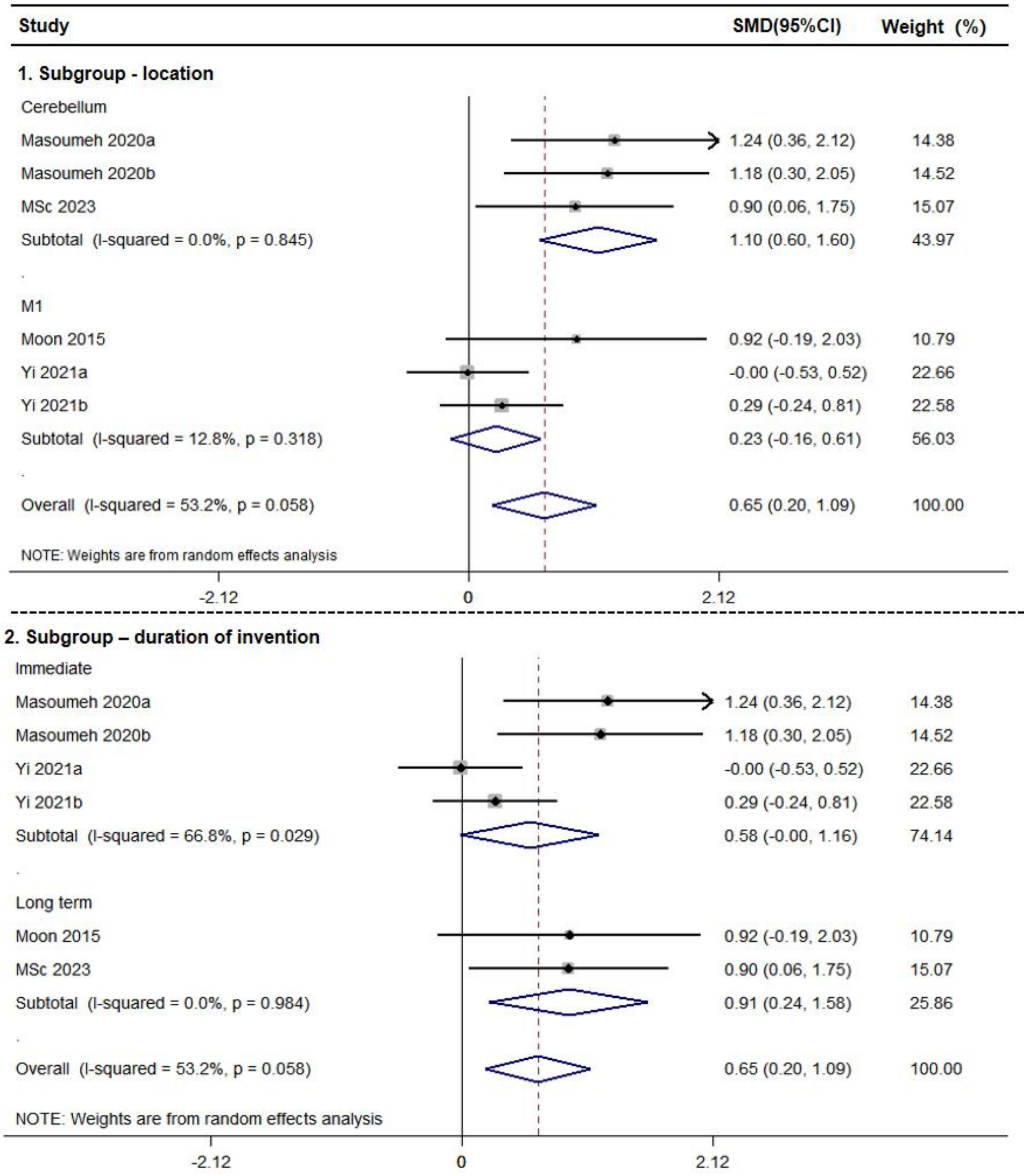

Figure 4. The forest plot results of Static balance-OLST comparing tDCS to Con.

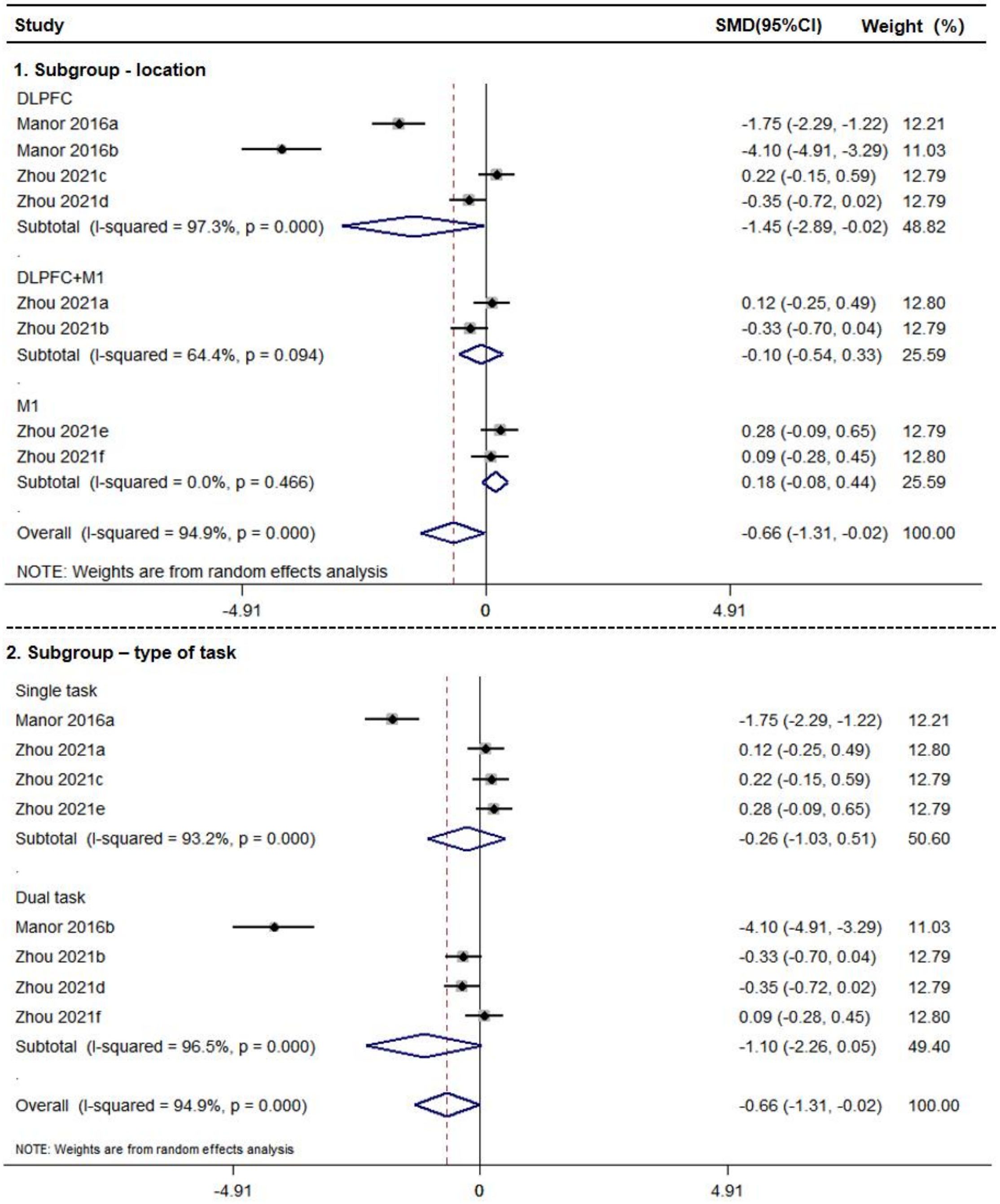

Figure 5. The forest plot results of Static balance-COP sway area comparing tDCS to Con.

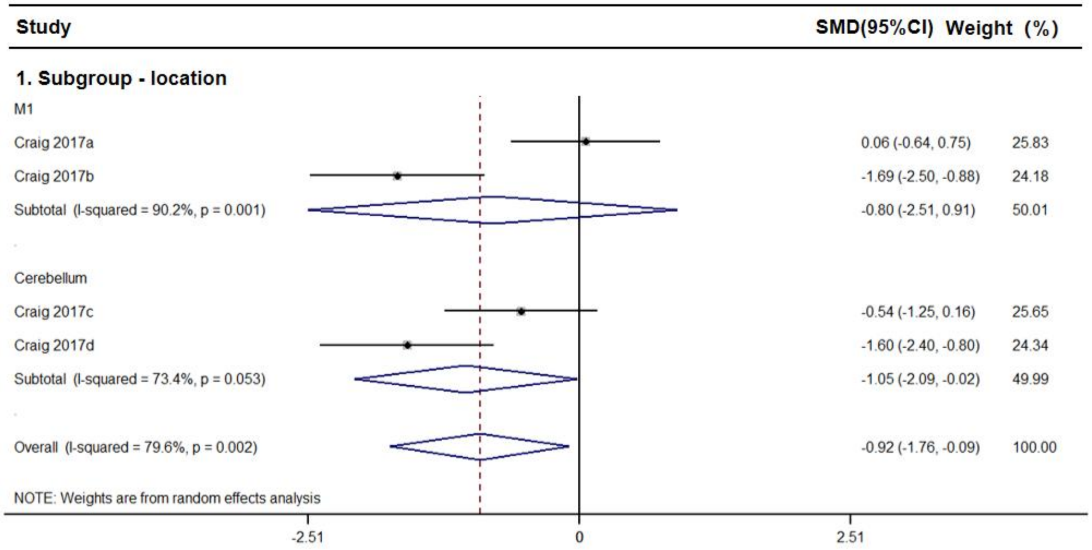

Figure 6. The forest plot results of Static balance-COP path length comparing tDCS to Con.

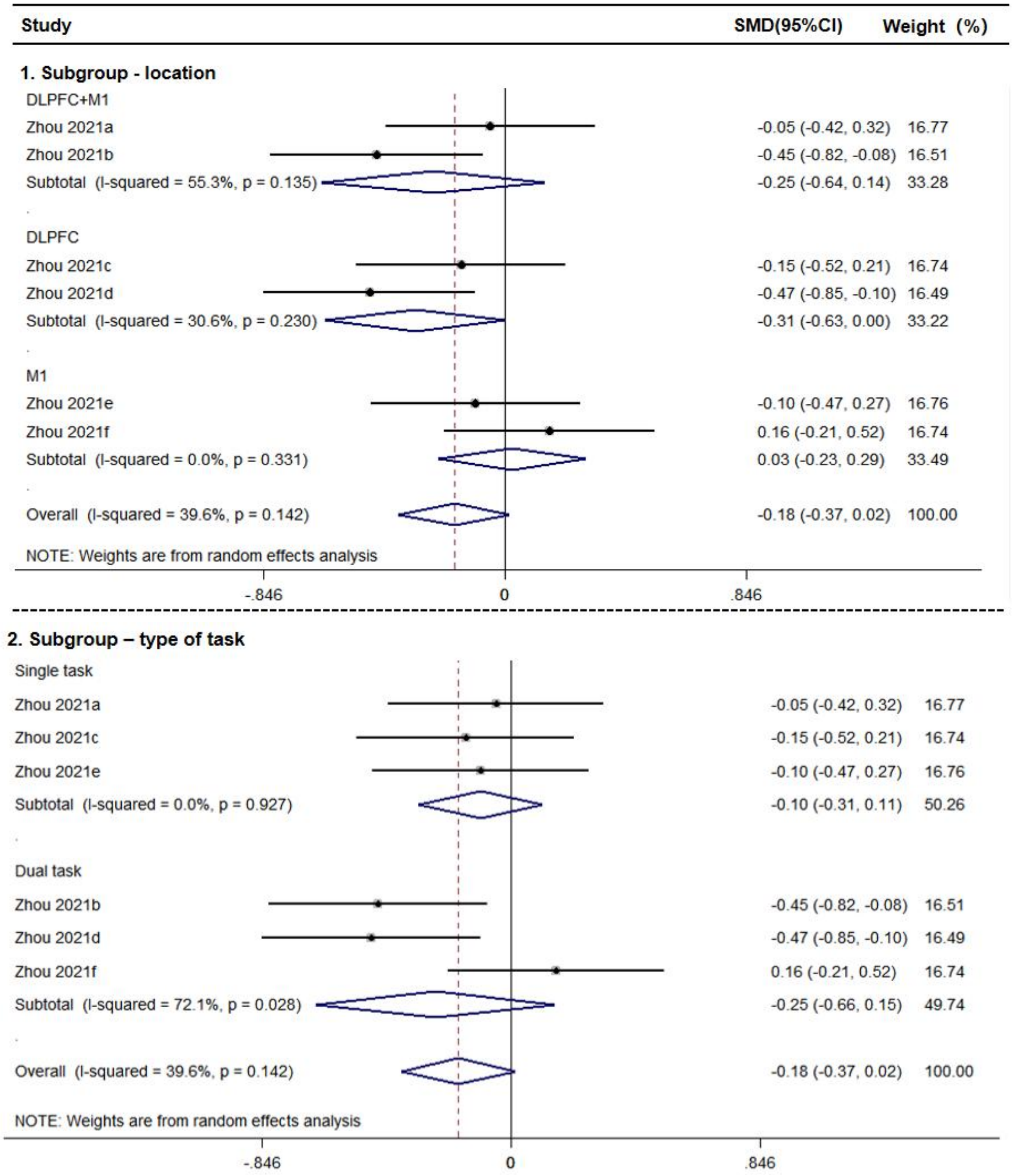

Figure 7. The forest plot results of Static balance-COP sway velocity comparing tDCS to Con.

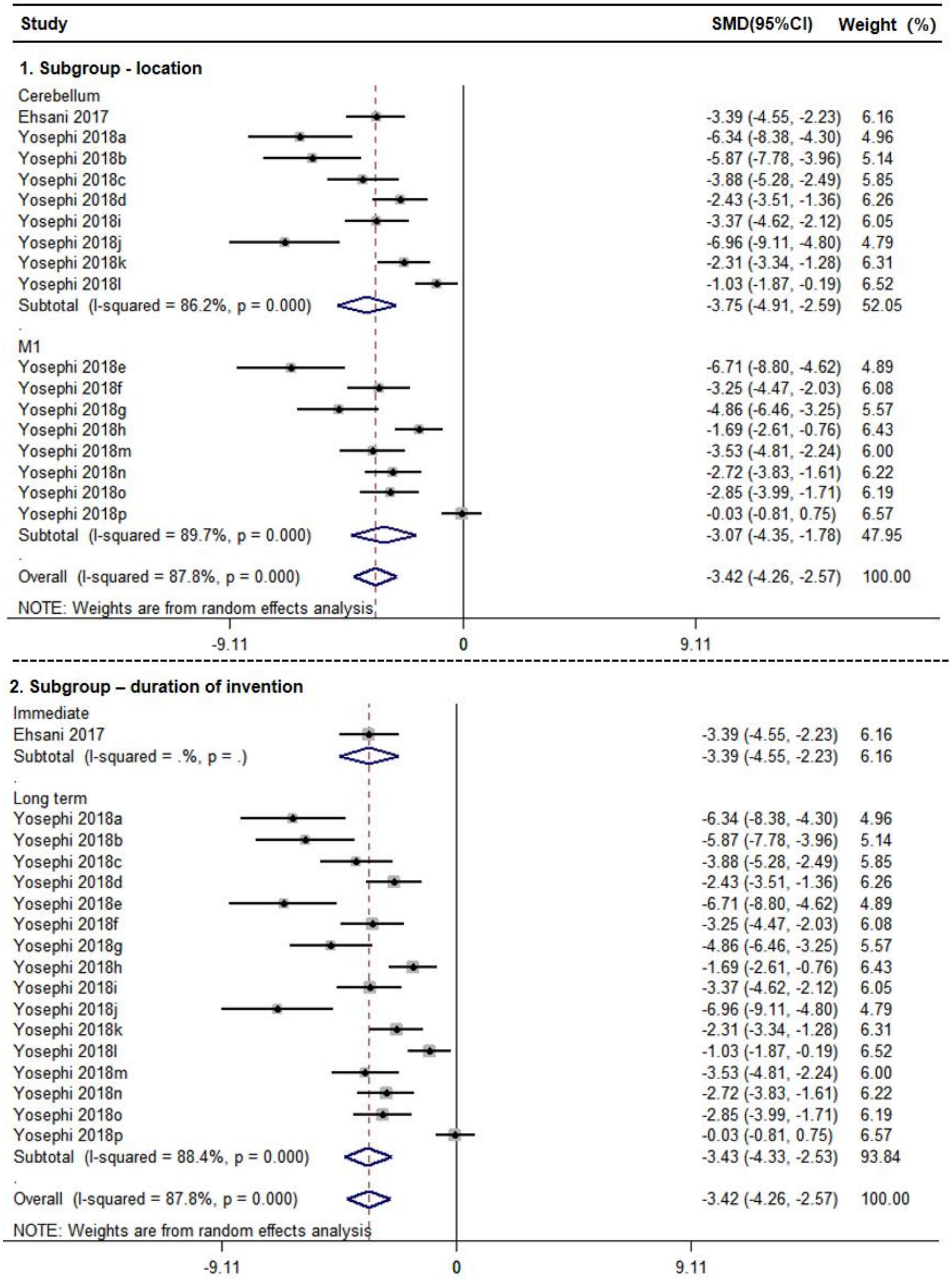

Figure 8. The forest plot results of Dynamic balance-APSI comparing tDCS to Con.

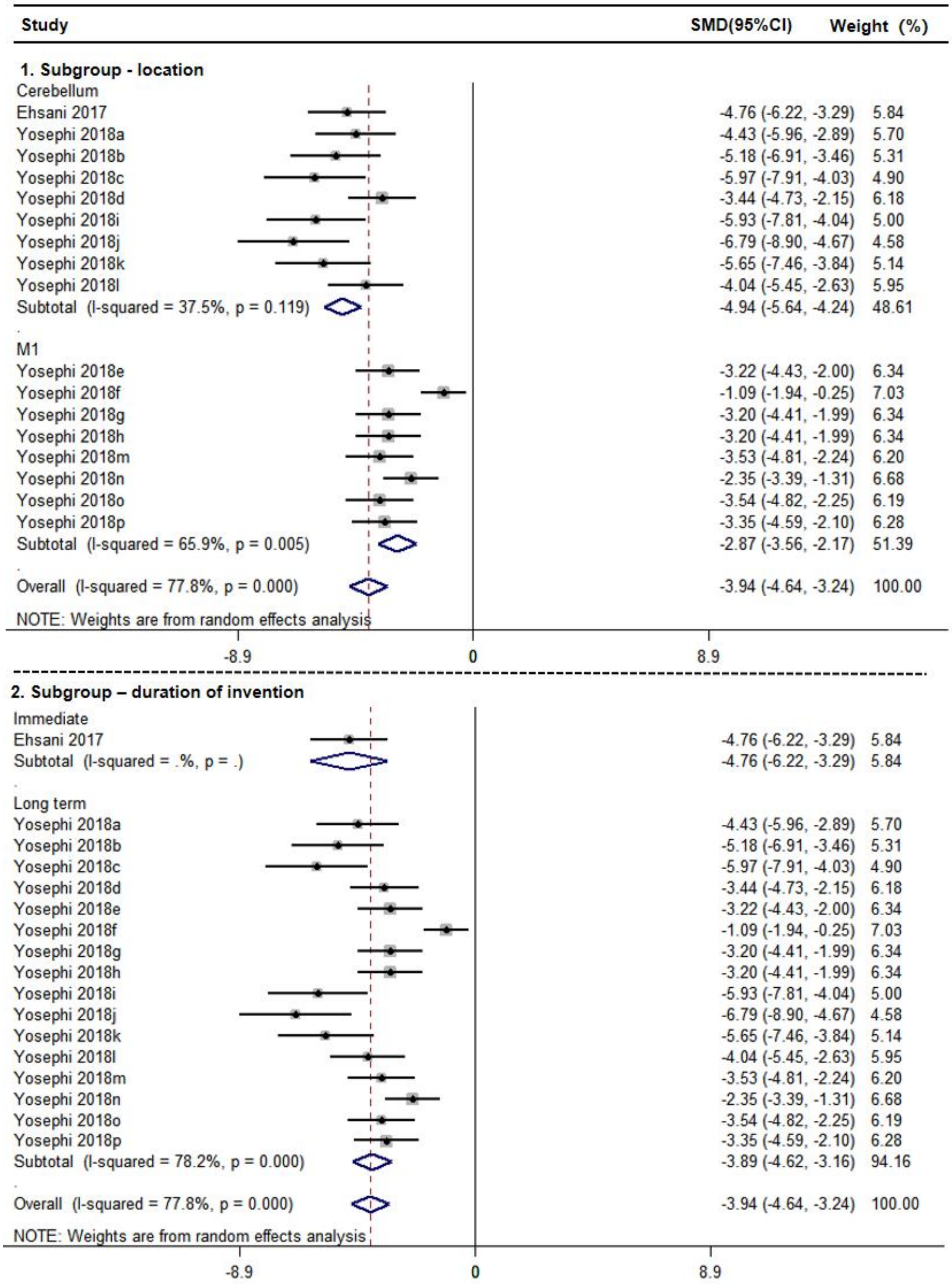

Figure 9. The forest plot results of Dynamic balance-MLSI comparing tDCS to Con.

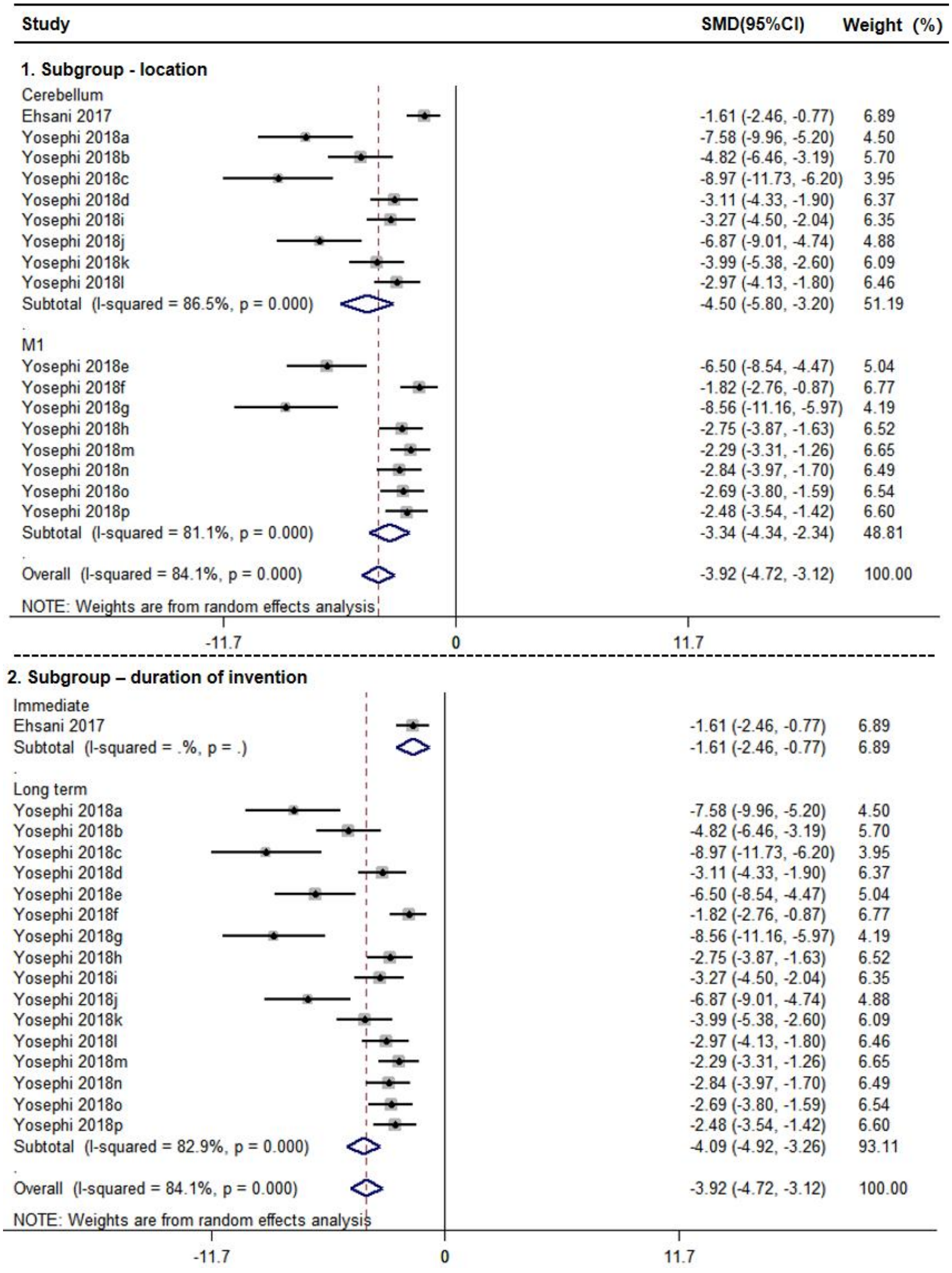

Figure 10. The forest plot results of Dynamic balance-OSI comparing tDCS to Con.

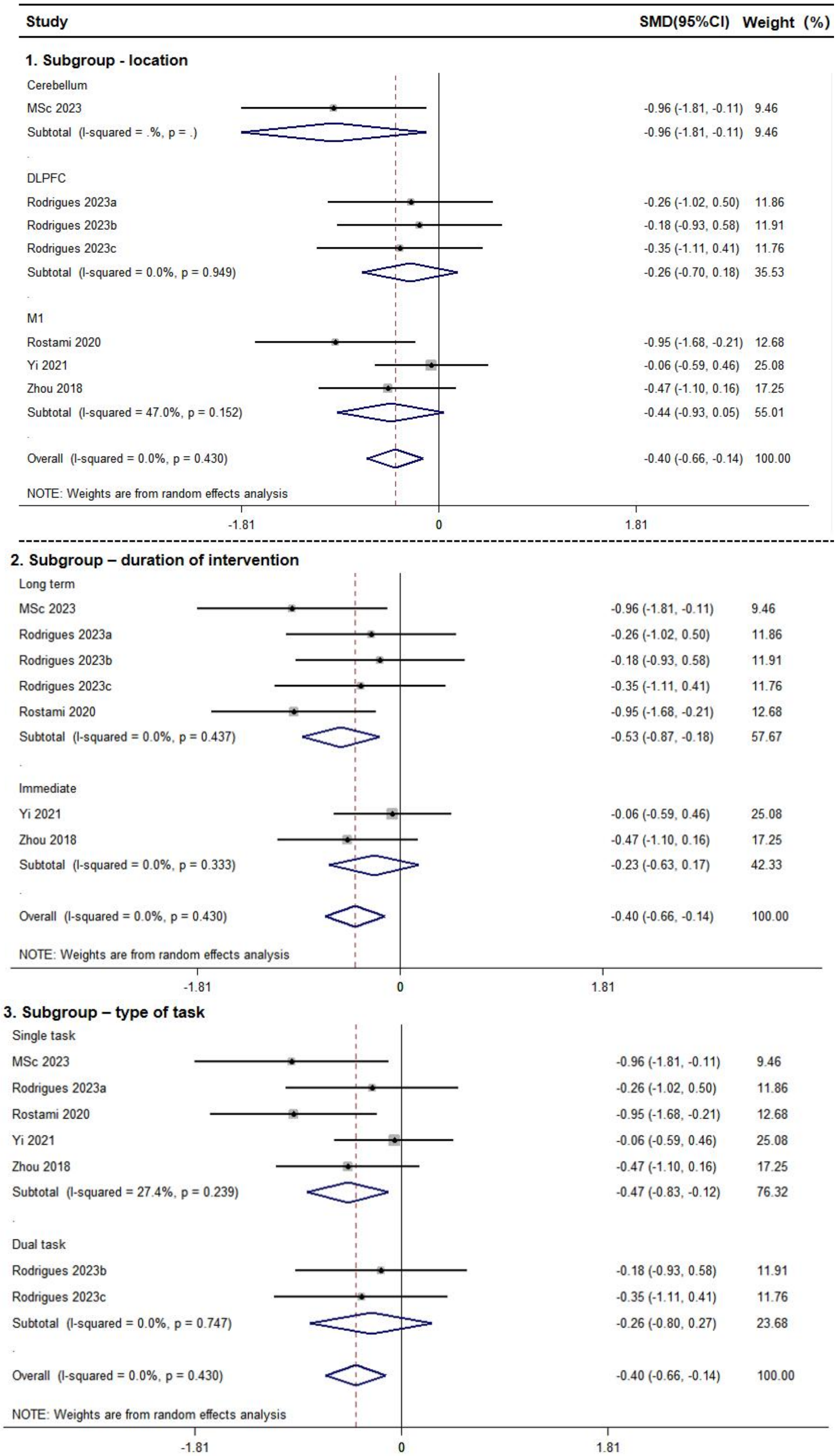

Figure 11. The forest plot results of Dynamic balance-TUGT comparing tDCS to Con.

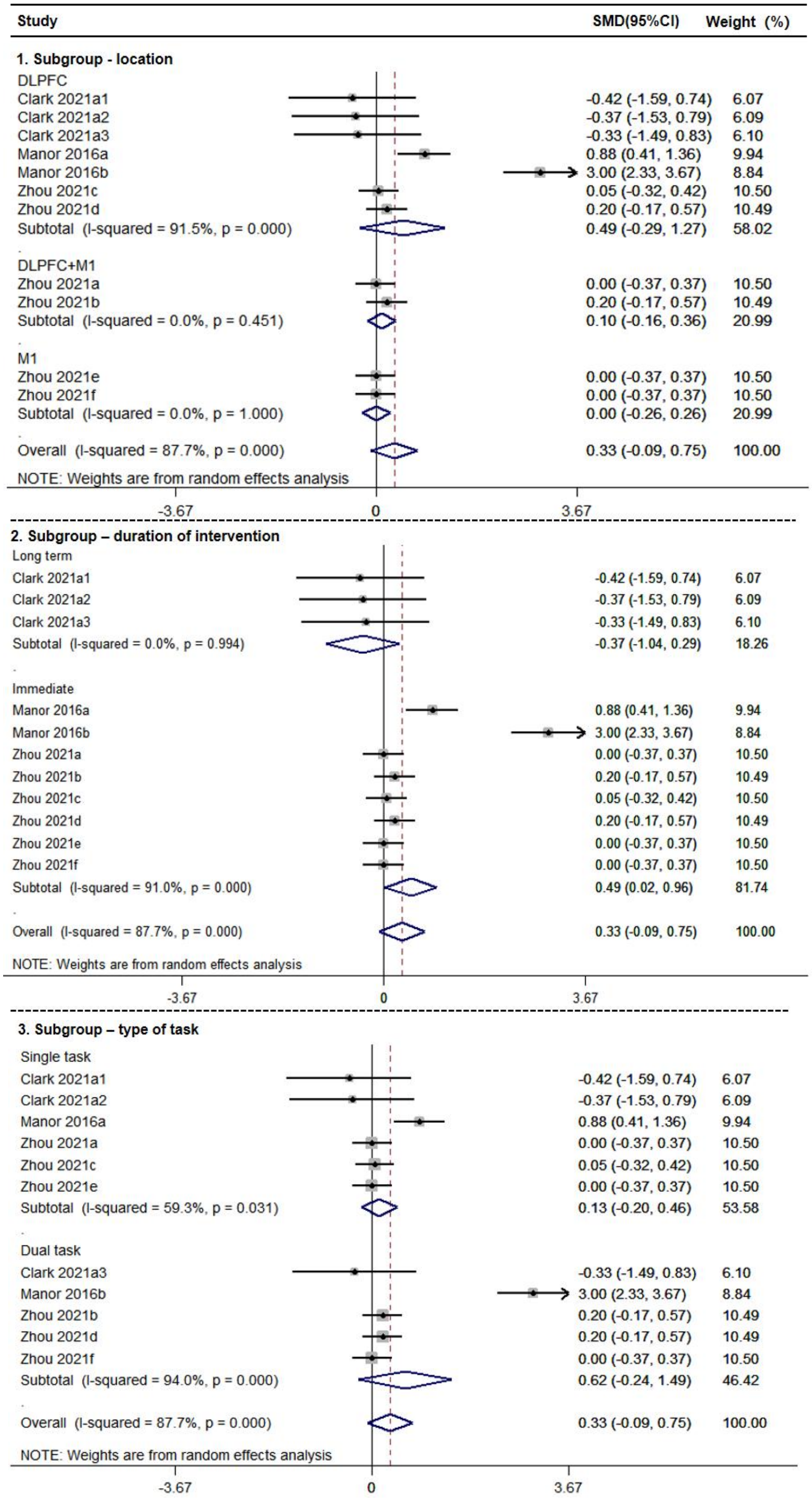

Figure 12. The forest plot results of Gait-walking speed comparing tDCS to Con.

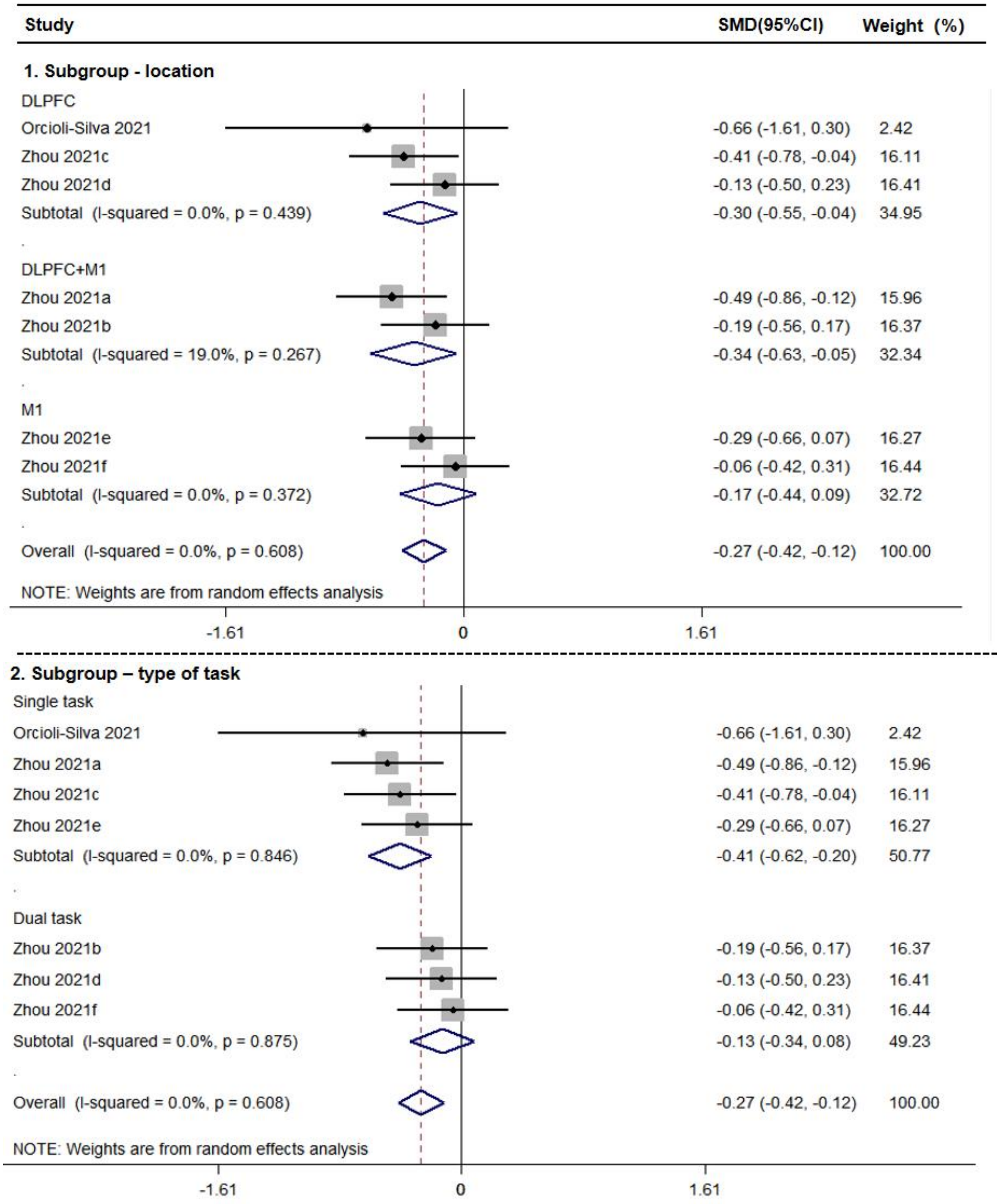

Figure 13. The forest plot results of Gait-stride time variability comparing tDCS to Con.

1.2 Funnel plots

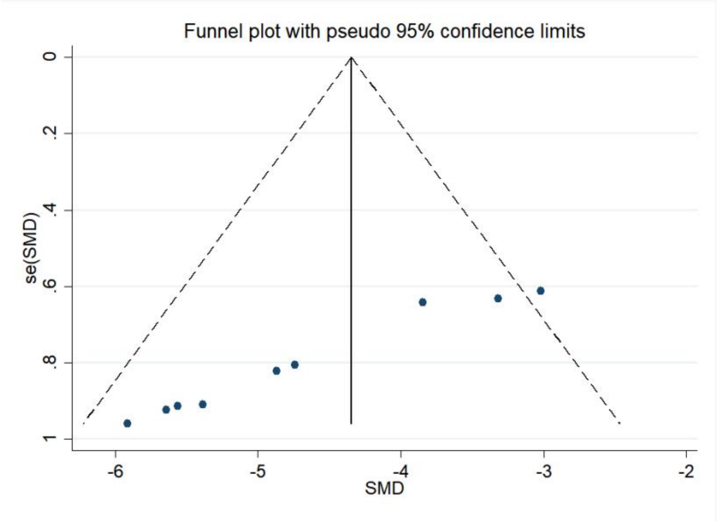

Figure 1. The funnel plot results of Static balance-APSI comparison between tDCS and Con.

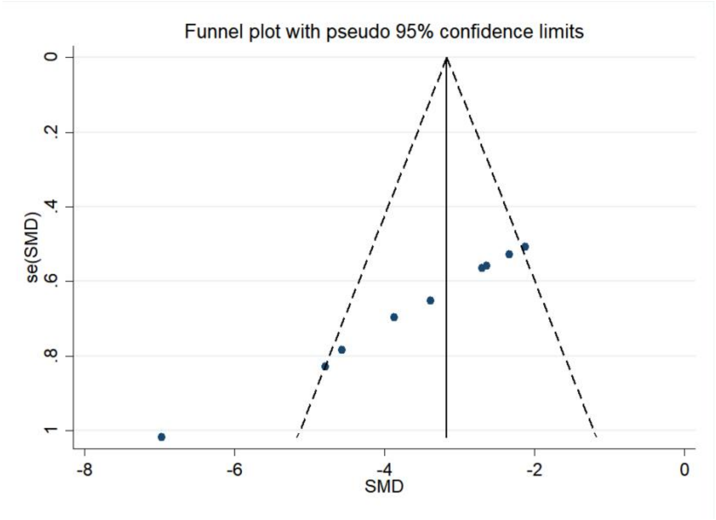

Figure 2. The funnel plot results of Static balance-MLSI comparison between tDCS and Con.

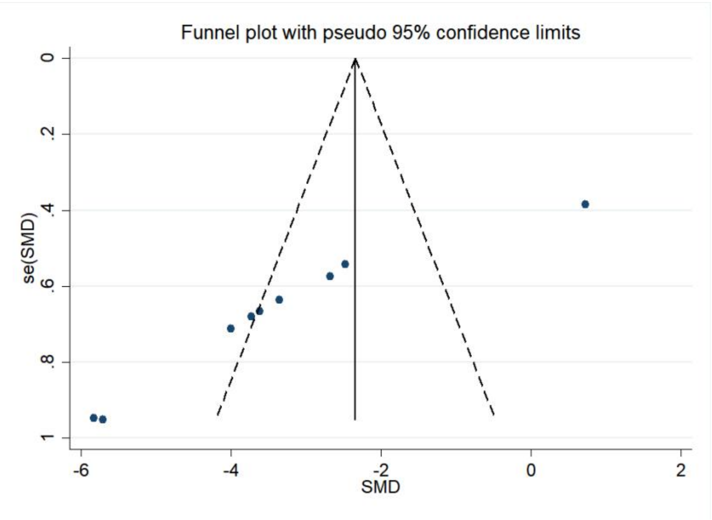

Figure 3. The funnel plot results of Static balance-OSI comparison between tDCS and Con.

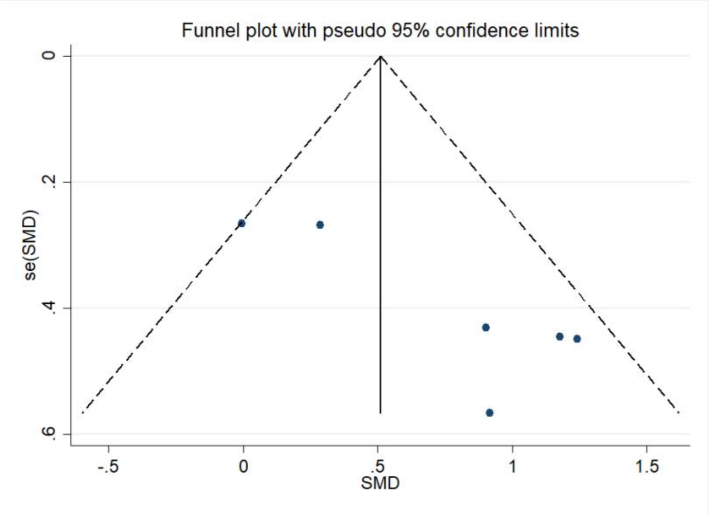

Figure 4. The funnel plot results of Static balance-OLST comparison between tDCS and Con.

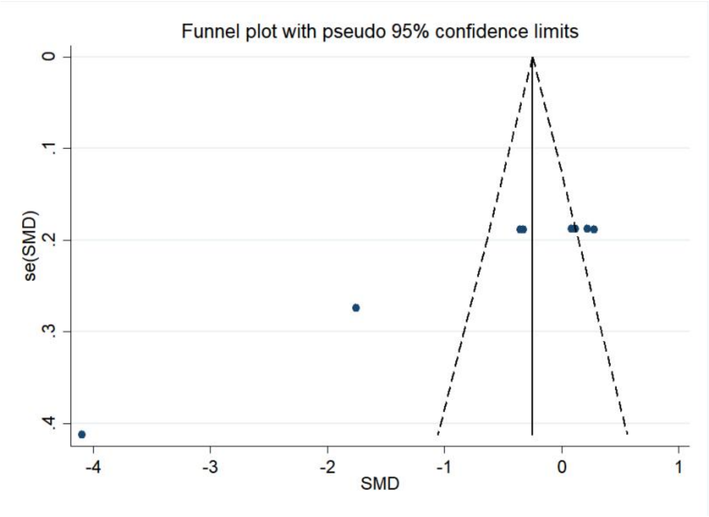

Figure 5. The funnel plot results of Static balance-COP sway area comparison between tDCS and Con.

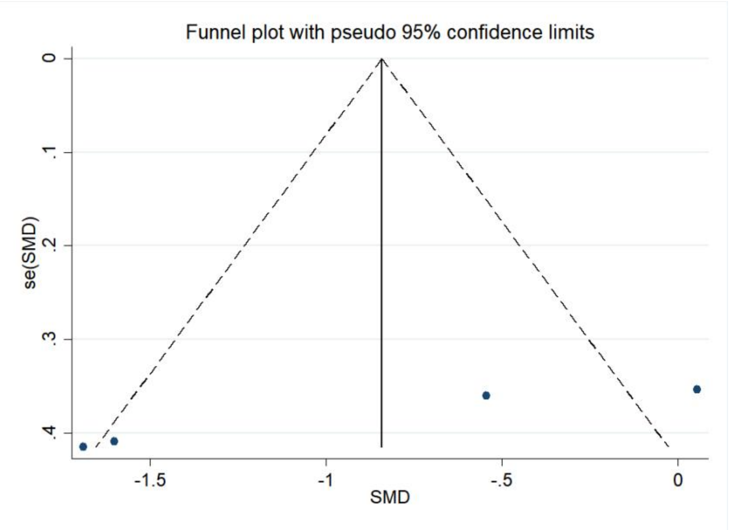

Figure 6. The funnel plot results of Static balance-COP path length comparison between tDCS and Con.

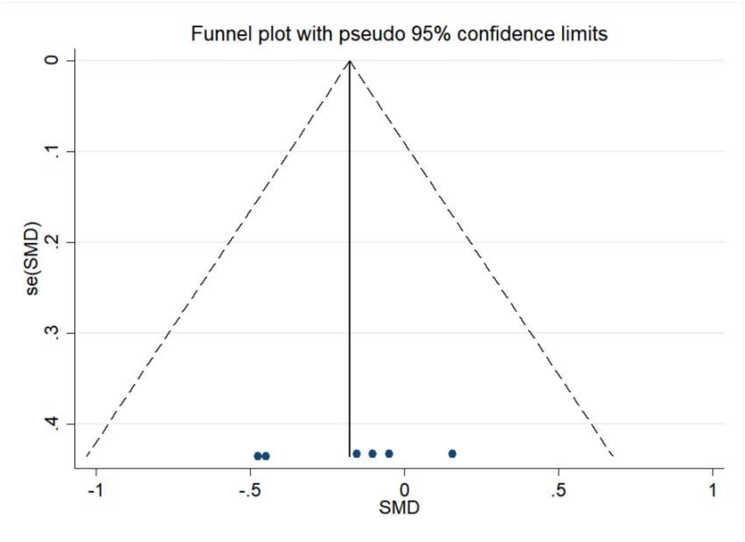

Figure 7. The funnel plot results of Static balance-COP sway velocity comparison between tDCS and Con.

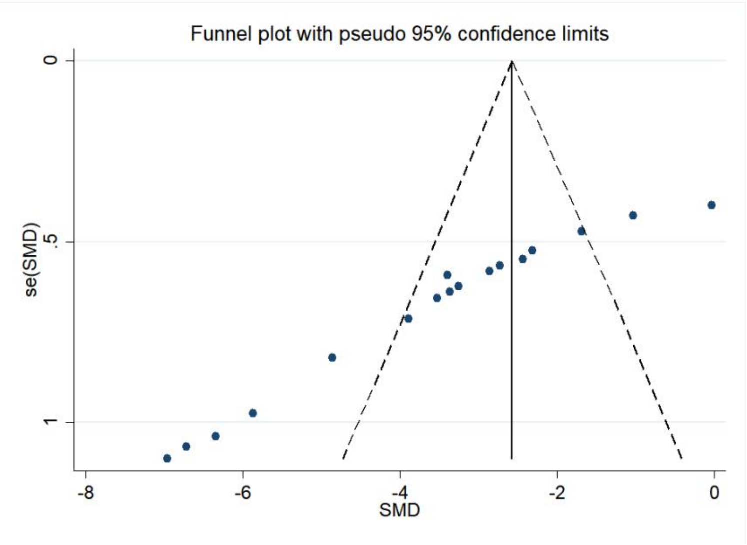

Figure 8. The funnel plot results of Dynamic balance-APSI comparison between tDCS and Con.

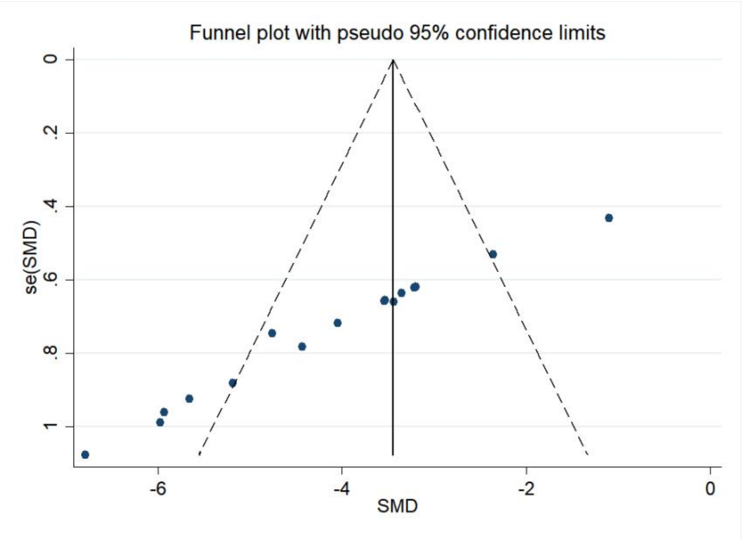

Figure 9. The funnel plot results of Dynamic balance-MLSI comparison between tDCS and Con.

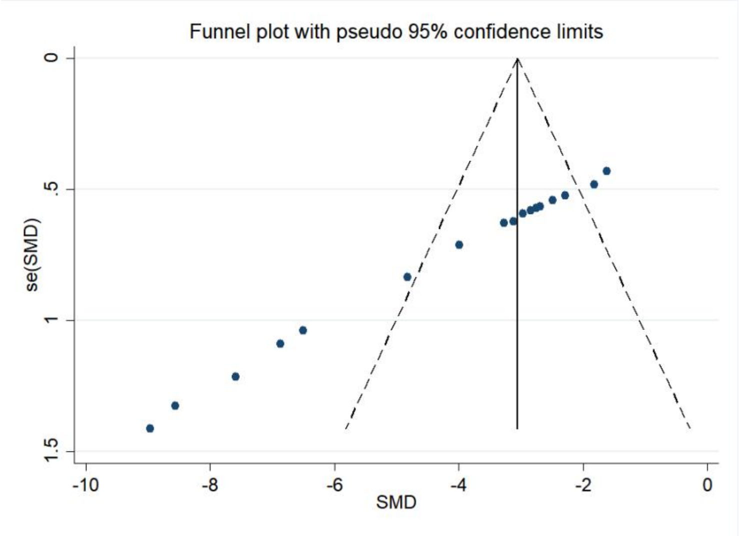

Figure 10. The funnel plot results of Dynamic balance-OSI comparison between tDCS and Con.

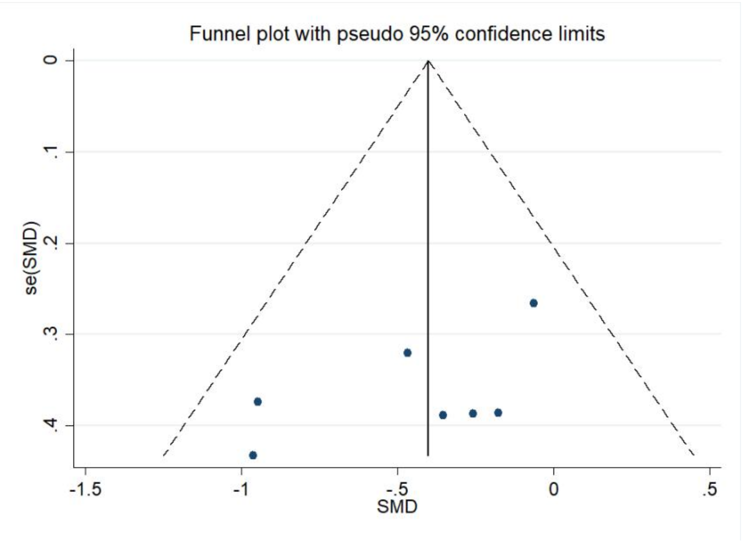

Figure 11. The funnel plot results of Dynamic balance-TUGT comparison between tDCS and Con.

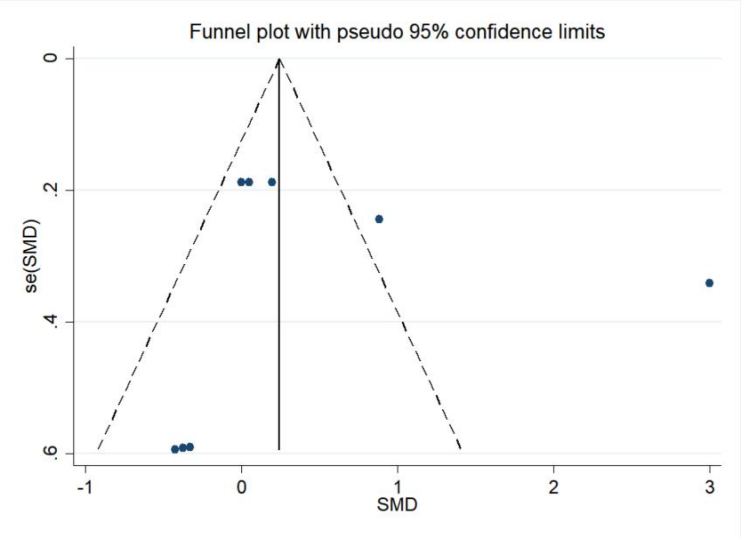

Figure 12. The funnel plot results of Gait-stride time variability comparison between tDCS and Con.

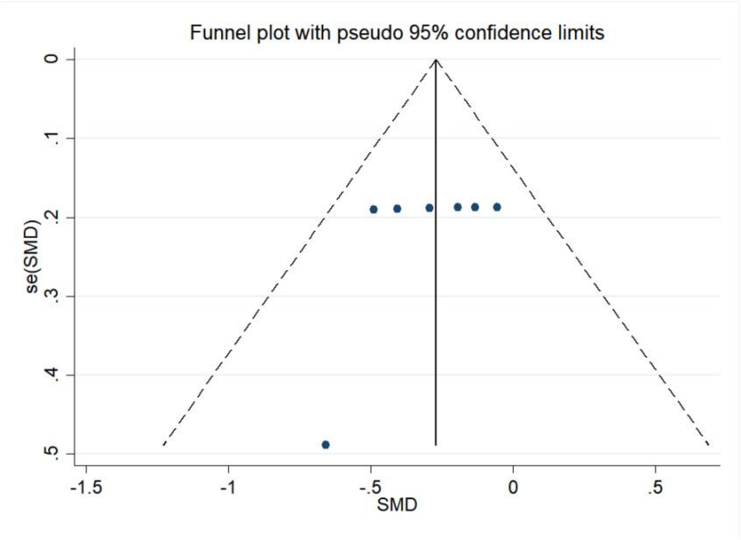

Figure 13. The funnel plot results of Gait-stride time variability comparison between tDCS and Con.

1.3 Sensitivity analysis

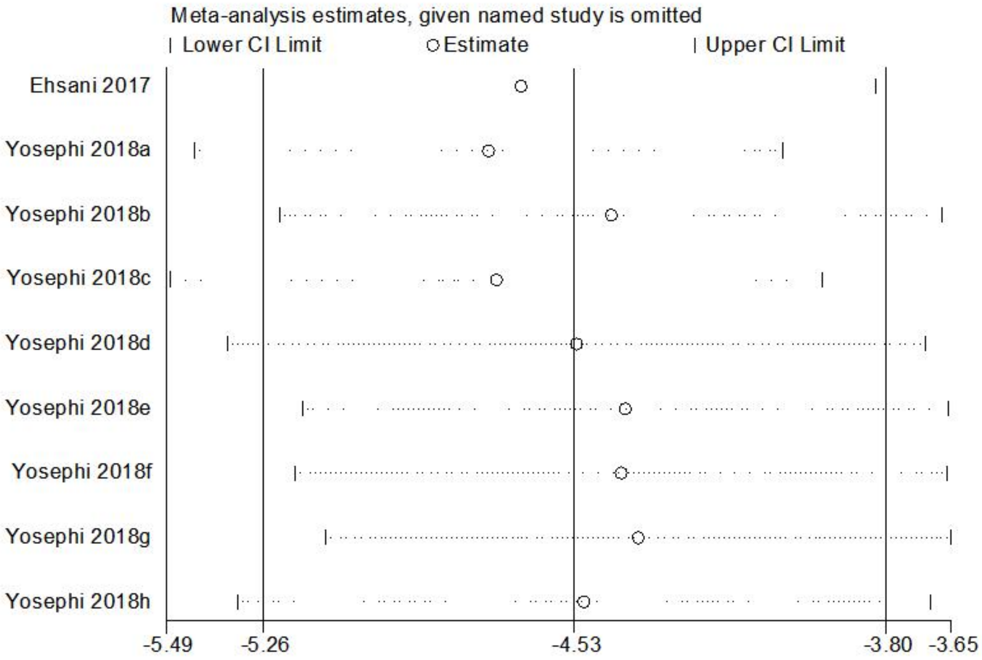

Figure 1. The sensitivity analysis results of Static balance-APSI comparison between tDCS and Con.

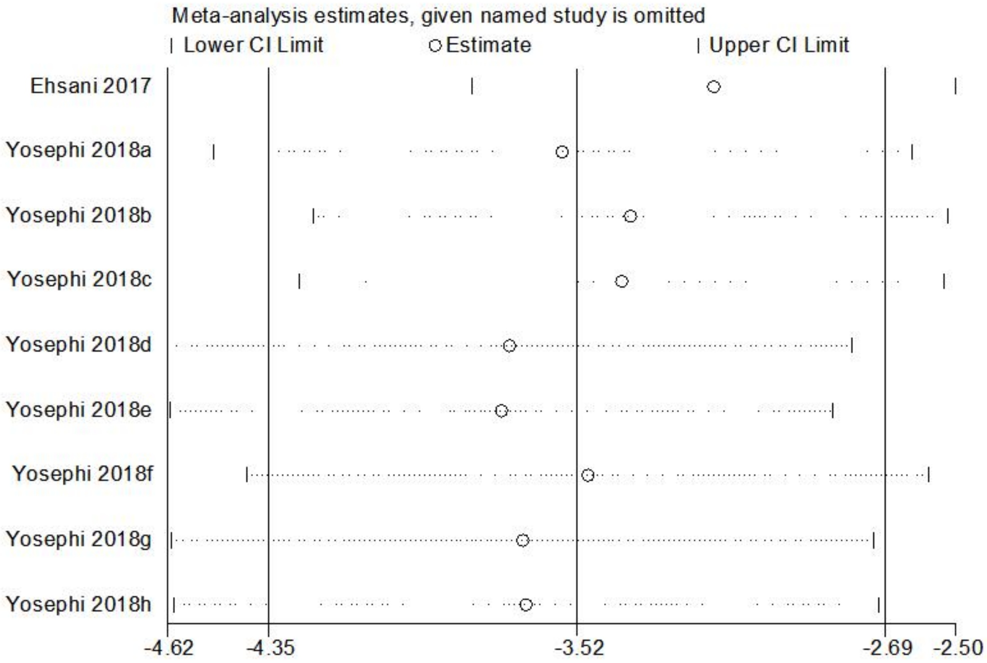

Figure 2. The sensitivity analysis results of Static balance-MLSI comparison between tDCS and Con.

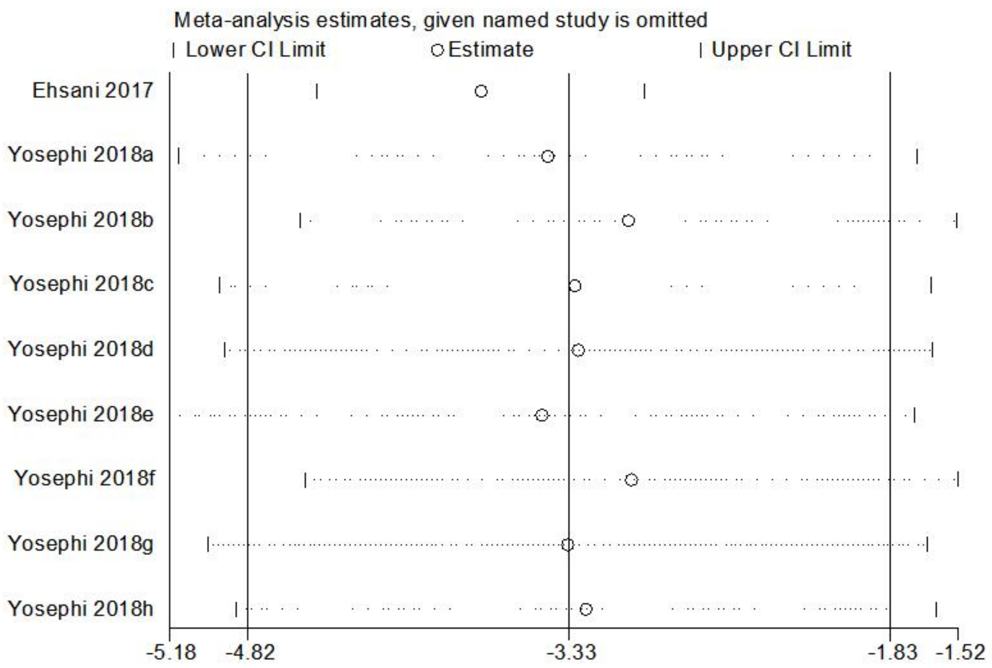

Figure 3. The sensitivity analysis results of Static balance-OSI comparison between tDCS and Con.

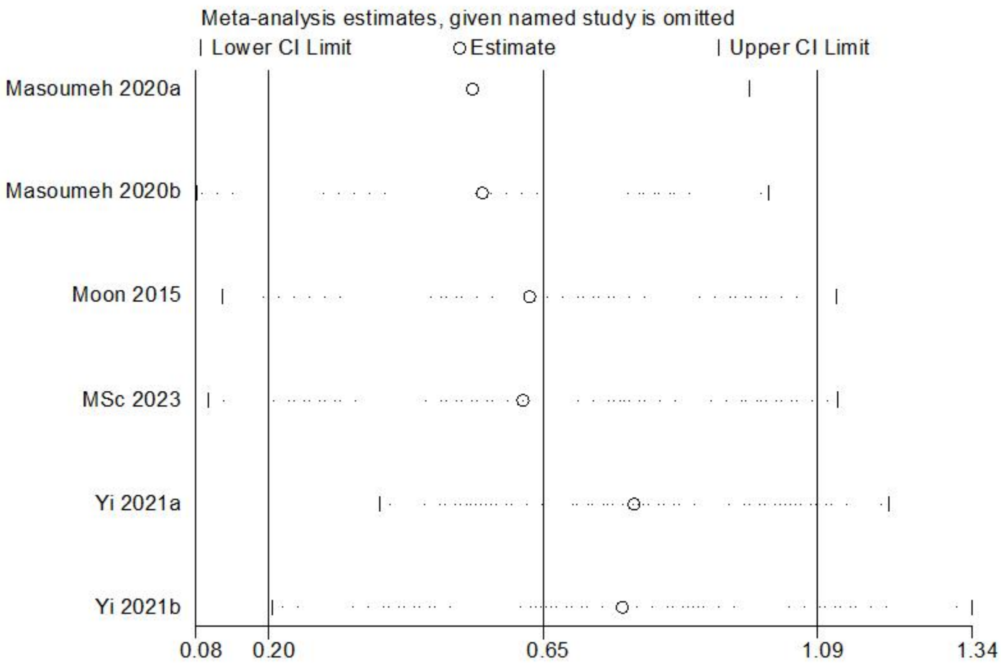

Figure 4. The sensitivity analysis results of Static balance-OLST comparison between tDCS and Con.

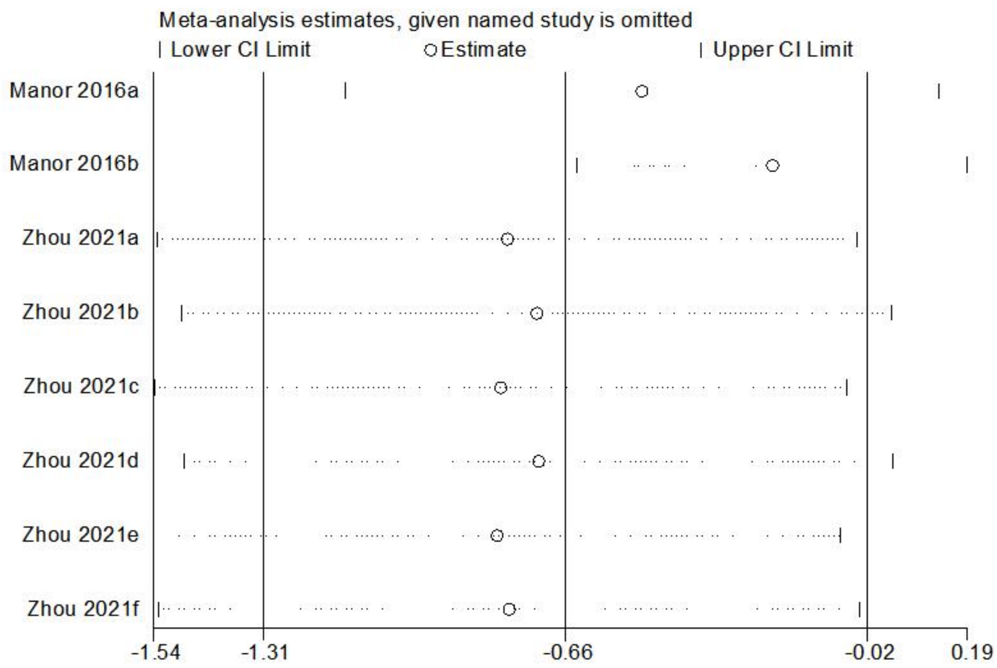

Figure 5. The sensitivity analysis results of Static balance-COP sway area comparison between tDCS and Con.

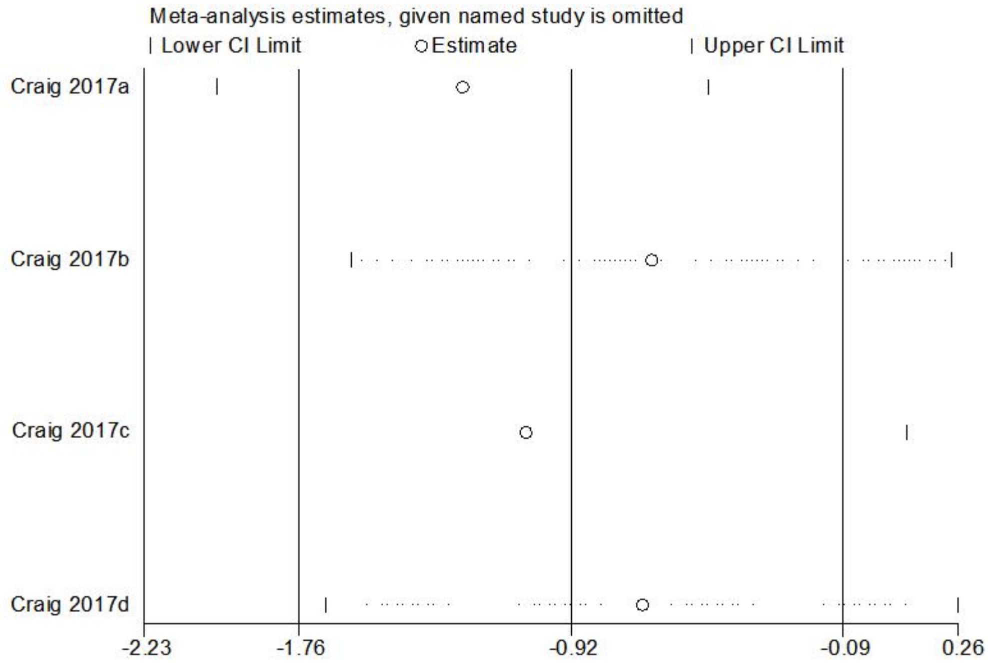

Figure 6. The sensitivity analysis results of Static balance-COP path length comparison between tDCS and Con.

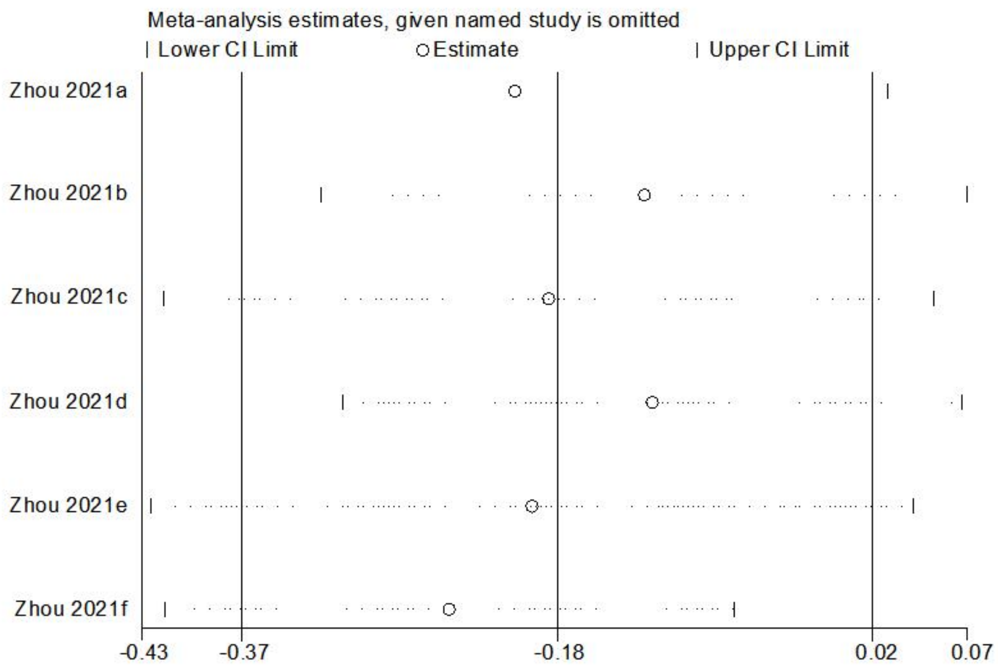

Figure 7. The sensitivity analysis results of Static balance-COP sway velocity comparison between tDCS and Con.

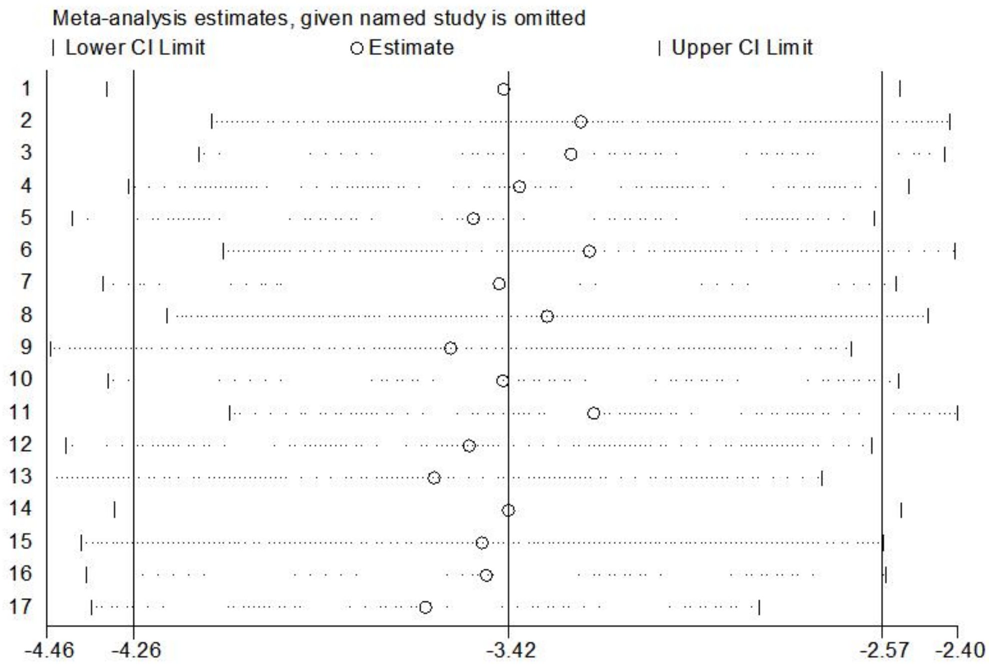

Figure 8. The sensitivity analysis results of Dynamic balance-APSI comparison between tDCS and Con.

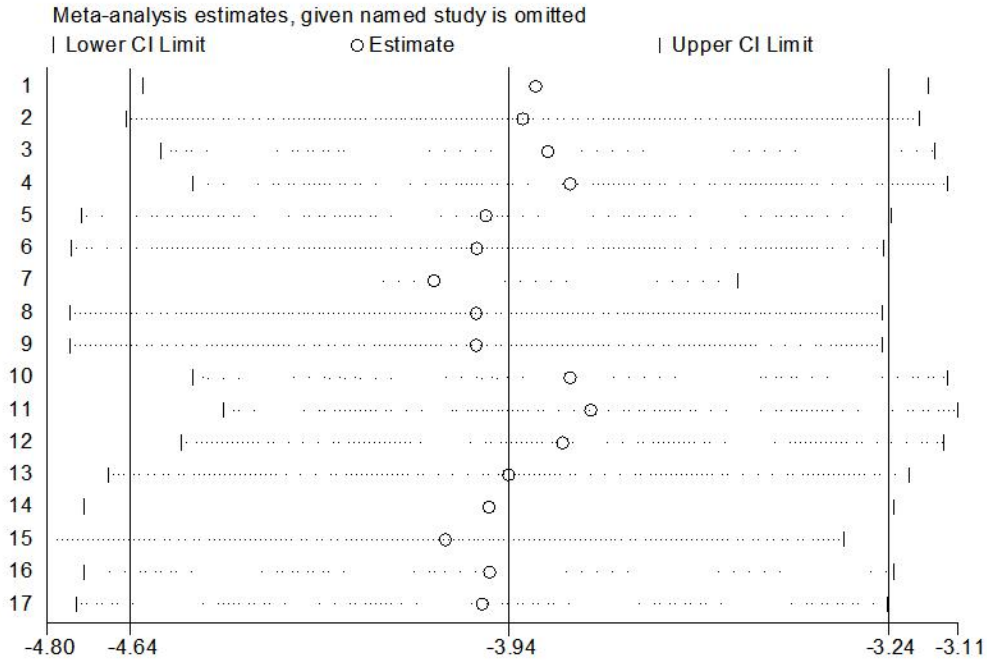

Figure 9. The sensitivity analysis results of Dynamic balance-MLSI comparison between tDCS and Con.

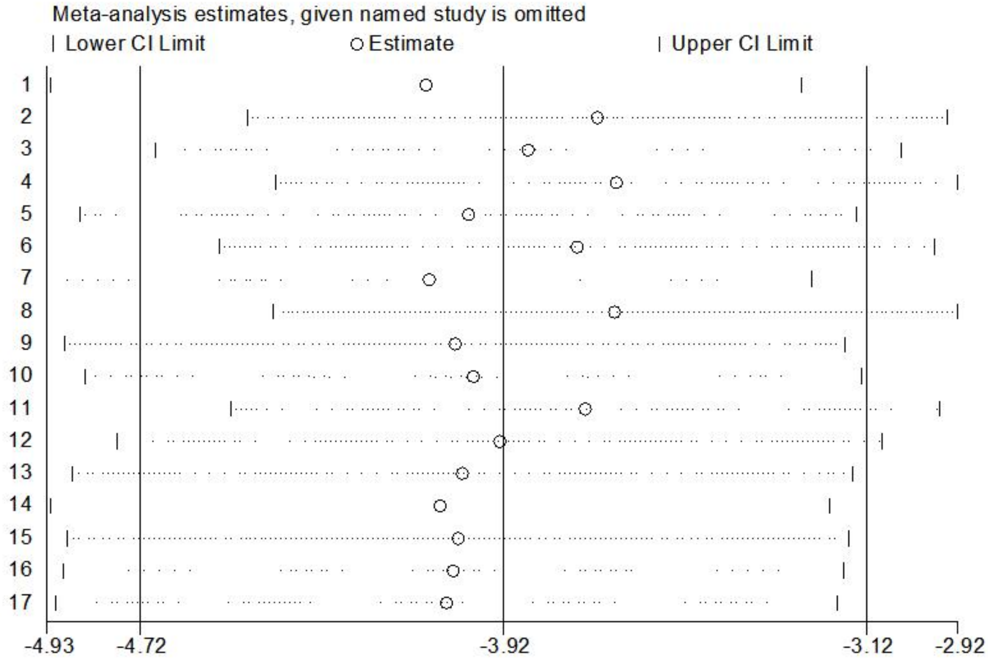

Figure 10. The sensitivity analysis results of Dynamic balance-OSI comparison between tDCS and Con.

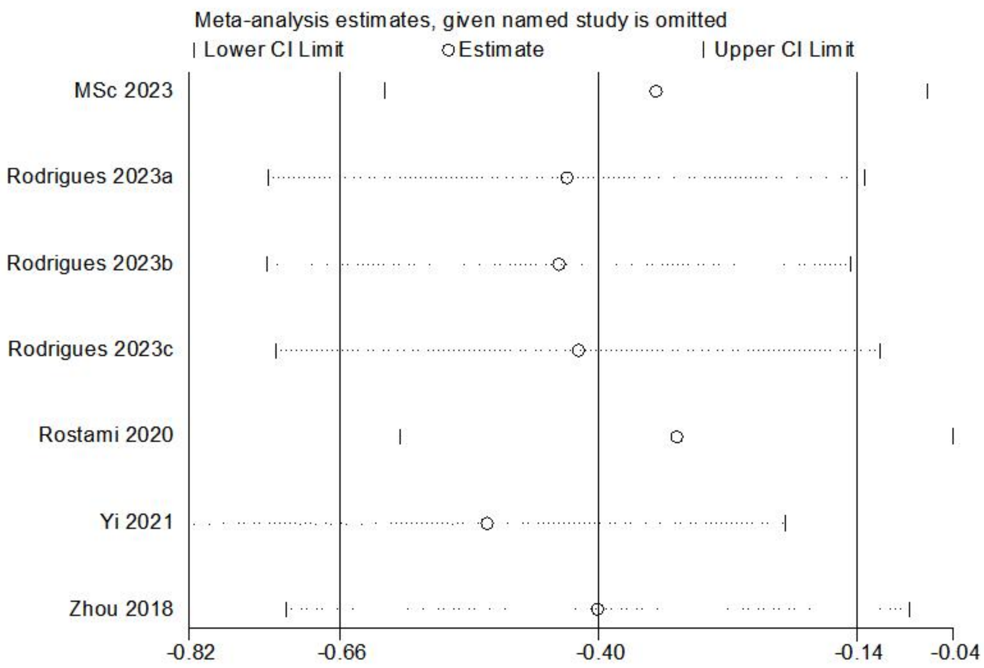

Figure 11. The sensitivity analysis results of Dynamic balance-TUGT comparison between tDCS and Con.

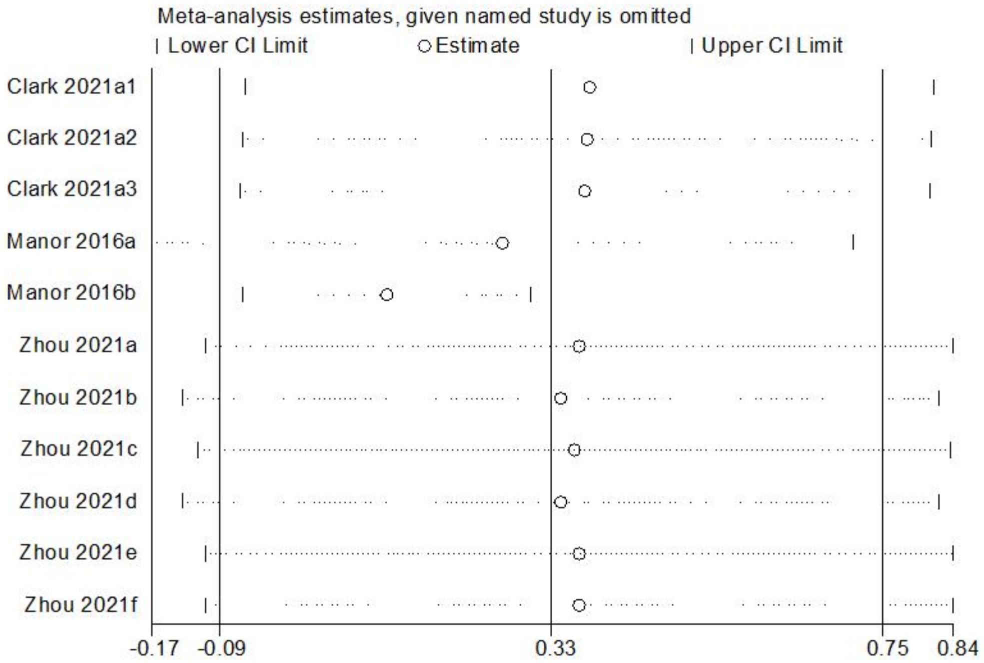

Figure 12. The sensitivity analysis results of Gait-stride time variability comparison between tDCS and Con.

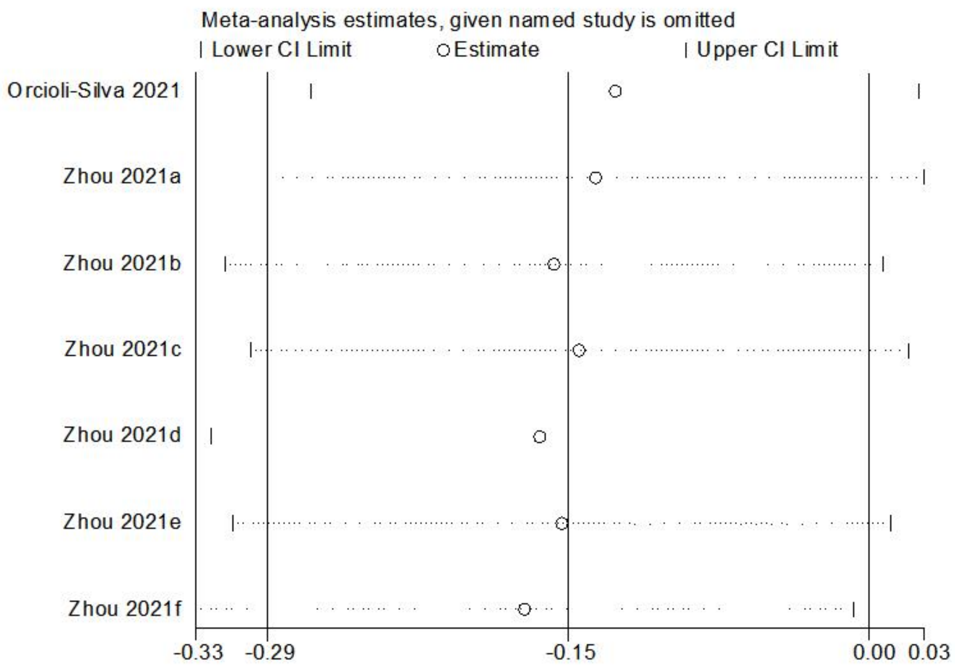

Figure 13. The sensitivity analysis results of Gait-stride time variability comparison between tDCS and Con.

2 Supplementary Tables

2.1 Search strategies

Table 1. Seach strategies in Pubmed.

Concept	Query	Hits
#1	("Transcranial Direct Current Stimulation"[Mesh]) OR (((((((tDCS[Title/Abstract]) OR (anodal stimulation transcranial direct current stimulation[Title/Abstract])) OR (anodal stimulation tDCS*[Title/Abstract])) OR (cathodal stimulation transcranial direct current stimulation[Title/Abstract])) OR (Cathodal Stimulation tDCS*[Title/Abstract])) OR (Transcranial Electrical Stimulation*[Title/Abstract])) OR (non-invasive brain stimulation[Title/Abstract]))	11474
#2	("Aged"[Mesh]) OR (((((aging[Title/Abstract]) OR (elderly[Title/Abstract])) OR (older adult*[Title/Abstract])) OR (old[Title/Abstract])) OR (older[Title/Abstract]))	5318409
#3	("Postural Balance"[Mesh]) OR ((((((((((((((((((((postural control*[Title/Abstract]) OR (balance[Title/Abstract])) OR (static balance[Title/Abstract])) OR (dynamic balance[Title/Abstract])) OR (postural stability[Title/Abstract])) OR (postural instability[Title/Abstract])) OR (posture equilibrium*[Title/Abstract])) OR (postural sway[Title/Abstract])) OR (stand*[Title/Abstract])) OR (stance[Title/Abstract])) OR (mobility[Title/Abstract])) OR (walk*[Title/Abstract])) OR (gait[Title/Abstract])) OR (sit-to-stand[Title/Abstract])) OR (STS[Title/Abstract])) OR (timed up[Title/Abstract] AND go[Title/Abstract])) OR (TUG[Title/Abstract])) OR (six min walk*[Title/Abstract])) OR (6MW[Title/Abstract])) OR (6 MWT[Title/Abstract]))	2611966
#4	#1 AND #2 AND #3	316

Table 2. Seach strategies in Cochrane Library.

Concept	Query	Hits
#1	(Transcranial Direct Current Stimulation):ti,ab,kw OR (tDCS):ti,ab,kw OR (anodal transcranial direct current stimulation):ti,ab,kw OR (anodal stimulation tDCS*):ti,ab,kw OR (cathodal transcranial direct current stimulation):ti,ab,kw OR (Cathodal Stimulation tDCS*):ti,ab,kw OR (Transcranial Electrical Stimulation*):ti,ab,kw OR (non-invasive brain stimulation):ti,ab,kw	9257
#2	(aged):ti,ab,kw OR (aging):ti,ab,kw OR (elderly):ti,ab,kw OR (older adult*):ti,ab,kw OR (old):ti,ab,kw OR (older):ti,ab,kw	787052
#3	(Postural Balance):ti,ab,kw OR (postural control*):ti,ab,kw OR (balance):ti,ab,kw OR (static balance):ti,ab,kw OR (dynamic balance):ti,ab,kw OR (postural stability):ti,ab,kw OR (postural instability):ti,ab,kw OR (posture equilibrium*):ti,ab,kw OR (postural sway):ti,ab,kw OR (stand*):ti,ab,kw OR (stance):ti,ab,kw OR (mobility):ti,ab,kw OR (walk*):ti,ab,kw OR (gait):ti,ab,kw OR (sit-to-stand):ti,ab,kw OR (STS):ti,ab,kw OR (timed up and go):ti,ab,kw OR (TUG):ti,ab,kw OR (six min walk*):ti,ab,kw OR (6MW):ti,ab,kw OR (6 MWT):ti,ab,kw	387035
#4	#1 AND #2 AND #3	**642**

Table 3. Seach strategies in Embase.

Concept	Query	Hits
#1	'transcranial direct current stimulation':ti,ab,kw OR tdcs:ti,ab,kw OR 'anodal transcranial direct current stimulation':ti,ab,kw OR 'anodal stimulation tdcs*':ti,ab,kw OR 'cathodal transcranial direct current stimulation':ti,ab,kw OR 'cathodal stimulation tdcs*':ti,ab,kw OR 'transcranial electrical stimulation*':ti,ab,kw OR 'non-invasive brain stimulation':ti,ab,kw	15852
#2	'aged':ti,ab,kw OR aging:ti,ab,kw OR elderly:ti,ab,kw OR 'older adult*':ti,ab,kw OR old:ti,ab,kw OR older:ti,ab,kw	4084458
#3	'postural balance':ti,ab,kw OR 'postural control*':ti,ab,kw OR balance:ti,ab,kw OR 'static balance':ti,ab,kw OR 'dynamic balance':ti,ab,kw OR 'postural stability':ti,ab,kw OR 'posture equilibrium*':ti,ab,kw OR 'postural sway':ti,ab,kw OR stand*:ti,ab,kw OR stance:ti,ab,kw OR mobility:ti,ab,kw OR walk*:ti,ab,kw OR gait:ti,ab,kw OR 'sit to stand':ti,ab,kw OR sts:ti,ab,kw OR ('timed up':ti,ab,kw AND go:ti,ab,kw) OR tug:ti,ab,kw OR 'six min walk*':ti,ab,kw OR 6mw:ti,ab,kw OR '6 mwt':ti,ab,kw	3620427
#6	#1 AND #2 AND #3	**323**

Table 4. Seach strategies in Web of Science.

Concept	Query	Hits
#1	(((((((TS=(Transcranial Direct Current Stimulation)) OR TS=(tDCS)) OR TS=(anodal stimulation transcranial direct current stimulation)) OR TS=(anodal stimulation tDCS*)) OR TS=(cathodal stimulation transcranial direct current stimulation)) OR TS=(Cathodal Stimulation tDCS*)) OR TS=(Transcranial Electrical Stimulation*)) OR TS=(non-invasive brain stimulation)	30654
#2	(((((TS=(aged)) OR TS=(aging)) OR TS=(elderly)) OR TS=(older adult*)) OR TS=(old)) OR TS=(older)	14570685
#3	(((((((((((((((((((TS=(Postural Balance)) OR TS=(postural control*)) OR TS=(balance)) OR TS=(static balance)) OR TS=(dynamic balance)) OR TS=(postural stability)) OR TS=(postural instability)) OR TS=(posture equilibrium*)) OR TS=(postural sway)) OR TS=(stand*)) OR TS=(stance)) OR TS=(mobility)) OR TS=(walk*)) OR TS=(gait)) OR TS=(sit-to-stand)) OR TS=(STS)) OR TS=(timed up and go)) OR TS=(TUG)) OR TS=(six min walk*)) OR TS=(6MW)) OR TS=(6 MWT)	12711722
#4	#1 AND #2 AND #3	**1319**

Table 5. Seach strategies in Scopus.

Concept	Query	Hits
#1	(TITLE-ABS-KEY (transcranial AND direct AND current AND stimulation) OR TITLE-ABS-KEY (tdcs) OR TITLE-ABS-KEY (anodal AND stimulation AND transcranial AND direct AND current AND stimulation) OR TITLE-ABS-KEY (anodal AND stimulation AND tdcs*) OR TITLE-ABS-KEY (cathodal AND stimulation AND transcranial AND direct AND current AND stimulation) OR TITLE-ABS-KEY (cathodal AND stimulation AND tdcs*) OR TITLE-ABS-KEY (transcranial AND electrical AND stimulation*) OR TITLE-ABS-KEY (non-invasive AND brain AND stimulation))	23772
#2	(TITLE-ABS-KEY (aged) OR TITLE-ABS-KEY (aging) OR TITLE-ABS-KEY (elderly) OR TITLE-ABS-KEY (older AND adult*) OR TITLE-ABS-KEY (old) OR TITLE-ABS-KEY (older))	9689486
#3	(TITLE-ABS-KEY (postural AND balance) OR TITLE-ABS-KEY (postural AND control*) OR TITLE-ABS-KEY (balance) OR TITLE-ABS-KEY (static AND balance) OR TITLE-ABS-KEY (dynamic AND balance) OR TITLE-ABS-KEY (postural AND stability) OR TITLE-ABS-KEY (postural AND instability) OR TITLE-ABS-KEY (posture AND equilibrium*) OR TITLE-ABS-KEY (postural AND sway) OR TITLE-ABS-KEY (stand*) OR TITLE-ABS-KEY (stance) OR TITLE-ABS-KEY (mobility) OR TITLE-ABS-KEY (walk*) OR TITLE-ABS-KEY (gait) OR TITLE-ABS-KEY (sit-to-stand) OR TITLE-ABS-KEY (sts) OR TITLE-ABS-KEY (timed AND up AND go) OR TITLE-ABS-KEY (tug) OR TITLE-ABS-KEY (six AND min AND walk*) OR TITLE-ABS-KEY (6mw) OR TITLE-ABS-KEY (6 mwt))	7935642
#6	#1 AND #2 AND #3	**930**

Table 6. Seach strategies in CNKI.

Concept	Query	Hits
#1	经颅直流电刺激 + tDCS + 非侵入性脑刺激	
#2	老年人 + 老人	
#3	姿势控制 + 平衡 + 步态 + 步行 + 坐站测试 + 计时走	
#4	#1 AND #2 AND #3	8

2.2 Characteristics of included studies

Table 1. Characteristics of included studies.

Author, year, study design	Country, site	Intervention	Sample size	Age	Position, polarity	Intensity, frequency, duration	Outcomes	Main findings
**Clark 2021 RCT**	USA, NA	Active tDCS + Complex Walking/ Sham tDCS + Complex Walking/ Sham tDCS + Typical Walking	7 (2M, 5F)/ 5 (1M, 4F)/ 6 (2M, 4F)	75.4±5.8/ 70.6±5.2/ 73.7±7.6	DLPFC, Anodal	2 mA 20min/day 3 days/week, 6 weeks	Dynamic balance ability: walking function (typical/fastest/obstacle walk speed, figure-8 walk time)	All groups demonstrated gains in walking performance.
**Corrêa 2023a RCT**	Brazil, community	VGT/ VGT+anodic tDCS/ VGT+sham tDCS	19 (19F)/ 19 (19F)/ 19 (19F)	70±6/ 69±5/ 69±6	DLPFC, Anodal	2 mA 20min/day 2 days/week, 4 weeks	Dynamic balance ability: Mini-BEST Test	There was an improvement in the postural balance of the three training groups that were independent of tDCS.
**Corrêa 2023b RCT**	Brazil, community	Active-tDCS/ Sham-tDCS	14 (1M, 13F)/ 14 (2M, 12F)	65±5/ 69±4	DLPFC, Anodal	2 mA 20min/day 2 days/week, 12 weeks	Dynamic balance ability: 6-minute walk test; Mini-BEST Test	Compared with the sham group, the anodic group showed a more significant improvement.
**Craig 2017 Cross-over**	UK, NA	M1 tDCS/ Cerebellar tDCS/ Sham tDCS	16 (6M, 10F)/ 16 (4M, 12F)	72.44 ± 4.03	M1/ Cerebellar, Anodal	2 mA 20min Immediate	Static and dynamic balance ability: COP trajectories in 6 conditions (Anterior-posterior path length)	There was no significant difference between the groups.
**Ehsani 2017 RCT**	Iran, NA	Active-tDCS/ Sham-tDCS	14 (6M, 8F)/ 15 (7M, 8F)	66.08± 6.33/ 65.50± 6.14	Cerebellar, Anodal	1.5 mA 20min Immediate	Static and dynamic balance ability: Berg Balance Scale; APSI; MLSI; OSI	There was significant improvement of postural balance in A-tDCS compared with Con.
**Kaminski 2017 RCT**	Germany, NA	Anodal -tDCS/ Sham tDCS	15/ 15	66.8 ± 5.63/ 68.6 ± 6.00	M1, Anodal	1 mA 20min Immediate	Dynamic balance ability: DBT-mean balance time	There was no significant difference between the groups.
**Lo 2023 RCT**	USA, supportive housing facilities	tDCS+PT/ Sham tDCS+PT	2 (1M, 1F)/ 4 (4F)	85±4/ 90.75±3.42	DLPFC, Anodal	≤1.5 mA 20min 2 days/week, 5 weeks	Dynamic balance ability: single/dual-task gait speed; gait stride time variability; TUG; Berg Balance Scale	It is feasible and safe to add a 20-min stimulation session prior to their PTsessions in very old fallers.

**Manor 2016 Cross-over**	China, NA	Real tDCS/ Sham tDCS	37 (12M, 25F)	61 ± 5	DLPFC, Anodal	2 mA 20min Immediate	Static balance ability: single/dual task- standing postural sway area; Dynamic balance ability: single/dual task-walking speed	tDCS improved performance solely within dual-task conditions.
**Masoumeh 2020 RCT**	Iran, NA	Unilateral tDCS/ Bilateral tDCS/ Sham tDCS	12/ 12 / 12	69.08±2.84/ 68.91±2.57/ 69.16±1.58	Cerebellar, Anodal	2 mA 15min 3 days/week, 1 week	Static balance ability: single leg standing time	There was a statistically significant difference between the unilateral tDCS group and the control group and the bilateral tDCS group with the control group.
**Moon 2015 RCT**	Korea, nursing home	tDCS/ Sham tDCS	7(7F)/ 7(7F)	≥65	M1, Anodal	0.5 mA 15min 3 days/week, 5 weeks	Static balance ability: single leg standing time; FRT; Dynamic balance ability: gait stride length; step length	It was found that the effect of tDCS on balance and gait.
**MSc 2023 RCT**	Iran, the elderly care centers and retirement centers	Real tDCS/ Sham tDCS	12 (12M)/ 12 (12M)	69.08± 2.84/ 69.16 ± 1.58	Cerebellar, Anodal	2 mA 15min 3 days/week, 1 week	Static balance ability: single leg standing time; Dynamic balance ability: TUG	tDCS can improve static and dynamic balance in inactive older adults.
**Orcioli-Silva 2021 RCT**	UK, NA	Active-tDCS+walking/ Sham-tDCS+walking	10 (5M, 5F)/ 9 (2M, 7F)	66.0 ± 6.3/ 69.9 ± 4.8	DLPFC, Anodal	0.6 mA 20min Immediate	Dynamic balance ability: gait-cadence; Stance time; Stride time variability; Stride length variability	Negative correlations were observed in stride length variability following active tDCS+walking intervention.
**Rodrigues 2023 RCT**	Brazil, Community-Dwelling	tDCS/ Sham-tDCS/ Cognitive control	14 (6M, 8F)/ 13 (3M, 10F)/ 15 (5M, 10F)	71.3 ± 7.6/ 70.9 ± 7.6/ 72.6 ± 7.8	DLPFC, Anodal	2 mA 20min/day 2 days/week, 8 weeks	Dynamic balance ability: single/dual task-TUG	Compared with other groups, no significant improvement was observed in the tDCS group.
**Rostami 2020 RCT**	Iran, NA	Anodal -tDCS/ Sham tDCS	16/ 16	68.8	M1, Anodal	1 mA 20min/day 5 days	Dynamic balance ability: TUG; time-MFEWT; steps-MFEWT; 30-s CST	A-tDCS over the M1 for 5 consecutive days improves lower extremity functional performance in the healthy older participants.

**Sadati 2019 RCT**	Iran, Rehabilitation center	tDCS/ Sham tDCS	10/ 10	65.3 ± 2.5/ 65.4 ± 2.5	Cerebellar, Anodal	2 mA 20min/day 5days	Static and dynamic balance ability: posture control index in 6 conditions	tDCS of the cerebellum has significant effects on the postural control equilibrium variables in the first sensory condition absent vision and fixed support , and the fifth sensory condition with absent vision and sway support.
**Yi 2021 RCT**	Korea , P-Elderly Welfare Center	tDCS/ Sham tDCS	31 (10M, 21F)/ 26 (9M, 17F)	78.13 ± 4.76/ 78.77 ± 4.80	M1, Anodal	1.98 mA 20min Immediate	Static balance ability: one-leg standing right; one-leg standing left; Dynamic balance ability: 10MWT; 5STST;TUG	There were significant differences in 10MWT speed and static balance on the left side in tDCS group compared with sham tDCS group.
**Yosephi 2018 RCT**	Iran, NA	Cerebellar a-tDCSwith postural training/ Motor cortex a-tDCSwithpostural training/ Sham tDCS with postural training/ Postural training/ Cerebellar a-tDCS	11 (5M, 6F)/ 12 (6M, 6F)/ 12 (7M, 5F)/ 11 (5M, 6F)/ 11 (5M, 6F)	66.91 ± 4.39/ 64.17 ± 3.48/ 67.17 ± 4.91/ 65.27 ± 4.00/ 66.91 ± 3.46	M1, Anodal	2 mA 20min/day 3 days/week, 2 weeks	Static and dynamic balance ability: Berg Balance Scale; APSI; MLSI; OSI	Postural training with M1 or bilateral cerebellar a-tDCS, especially bilateral cerebellar a tDCS, can significantly improve postural control or balance in older adults with high fall risk.
**Zhou 2018 Cross-over**	China, NA	tDCS/ Sham tDCS	20	61 ± 4	SM1, Anodal	2 mA 20min Immediate	Dynamic balance ability: TUG	A trend towards better TUG performance following tDCS was observed.
**Zhou 2021 Cross-over**	USA, SeniorLife and Medical Center	L-DLPFC+SM1 tDCS/ L-DLPFC tDCS/ SM1 tDCS/ Sham tDCS	57 (12M, 45F)	75 ± 5	DLPFC+SM1 / DLPFC/ SM1, Anodal	1.5 mA 20min Immediate	Static balance ability: single/dual task-standing sway area;sway speed; Dynamic balance ability: single/dual task-gait speed; stride time variability	tDCS may be used to improve the ability of older adults to walk and stand under challenging conditions.

Notes: NA: not applicable; DLPFC: dorsolateral prefrontal cortex; M1:motor cortex; SM1: sensorimotor cortex; APSI:anterior-posterior stability index; MLSI:medial–lateral stability index; OSI:overall stability index; DBT:whole-body dynamic balancing task; TUG:timed up and go; FRT:functional stretch test; time-MFEWT:modified figure of 8 walk test time; steps-MFEWT:number of steps in modified figure of 8 walk test; 30-s CST:30-s chair stand test; 10MWT-10 meter walk test; 5STST:5-repetition sit-to-stand test.

2.3 Meta-regression

Table 1. The regression analysis results of tDCS on dynamic and static postural control in the elderly.

Covariate	β (95% Confidence Interval)	Standard Error	*P*-value
**Static balance-OLST**			
Publication year	-0.05 (-0.35, 0.25)	0.11	0.657
region	-	-	-
Proportion of males	-1.02 (-0.21, 2.26)	0.44	0.083
Mean age	-0.09 (-0.18, -0.01)	0.03	**0.041***
Stimulus intensity	0.24 (-0.62, 1.10)	0.31	0.479
**Dynamic balance-TUGT**			
Publication year	0.03 (-0.17, 0.23)	0.77	0.724
region	0.23 (-0.54, 1.01)	0.30	0.475
Proportion of males	-1.24 (-3.04, 0.52)	0.69	0.128
Mean age	0.03 (-0.03, 0.09)	0.02	0.251
Stimulus intensity	0.60 (-0.43, 1.24)	0.40	0.195

*Significant influence factors, 95% CI does not contain zero.

2.4 GRADE summary of evidence

Table 1. GRADE summary of evidence.

Outcome	Risk of bias	Inconsistency	Indirectness	Imprecision	Publication bias	Certainty of evidencee
Static balance-APSI	Serious	Serious	Not serious	Serious	Serious	Very low
Static balance-MLSI	Serious	Serious	Not serious	Serious	Serious	Very low
Static balance-OSI	Serious	Serious	Not serious	Serious	Serious	Very low
Static balance-OLST	Serious	Serious	Not serious	Serious	Serious	Very low
Static balance-COP sway area	Serious	Serious	Not serious	Serious	Serious	Very low
Static balance-COP path length	Serious	Serious	Not serious	Serious	Not serious	Very low
Static balance-COP sway velocity	Serious	Not serious	Not serious	Serious	Not serious	Low
Dynamic balance-APSI	Serious	Serious	Not serious	Serious	Serious	Very low
Dynamic balance-MLSI	Serious	Serious	Not serious	Serious	Serious	Very low
Dynamic balance-OSI	Serious	Serious	Not serious	Serious	Serious	Very low
Dynamic balance-TUGT	Serious	Not serious	Not serious	Serious	Not serious	Low
Gait-walking speed	Serious	Serious	Not serious	Serious	Serious	Low
Gait-Stride time variability	Serious	Not serious	Not serious	Serious	Not serious	Low
